# A Dual‐domain Engineered Antibody for Efficient HBV Suppression and Immune Responses Restoration

**DOI:** 10.1002/advs.202305316

**Published:** 2024-02-11

**Authors:** Yichao Jiang, Xiaoqing Chen, Xinya Ye, Can Wen, Tao Xu, Chao Yu, Wenjing Ning, Guosong Wang, Xinchu Xiang, Xiaomin Liu, Yalin Wang, Yuanzhi Chen, Xue Liu, Changrong Shi, Chao Liu, Quan Yuan, Yixin Chen, Tianying Zhang, Wenxin Luo, Ningshao Xia

**Affiliations:** ^1^ State Key Laboratory of Vaccines for Infectious Diseases Xiang An Biomedicine Laboratory School of Public Health School of Life Sciences Xiamen University Xiamen 361102 P.R. China; ^2^ State Key Laboratory of Vaccines for Infectious Diseases Center for Molecular Imaging and Translational Medicine Xiang An Biomedicine Laboratory School of Public Health Xiamen University Xiamen 361102 P.R. China; ^3^ State Key Laboratory of Molecular Vaccinology and Molecular Diagnostics National Institute of Diagnostics and Vaccine Development in Infectious Diseases National Innovation Platform for Industry‐Education Integration in Vaccine Research School of Public Health School of Life Sciences Xiamen University Xiamen 361102 P.R. China; ^4^ The Research Unit of Frontier Technology of Structural Vaccinology of Chinese Academy of Medical Sciences Xiamen University Xiamen 361102 P.R. China

**Keywords:** antibody‐based immunotherapy, chronic hepatitis B, immune restoration, therapeutic efficacy

## Abstract

Chronic hepatitis B (CHB) remains a major public health concern because of the inefficiency of currently approved therapies in clearing the hepatitis B surface antigen (HBsAg). Antibody‐based regimens have demonstrated potency regarding virus neutralization and HBsAg clearance. However, high dosages or frequent dosing are required for virologic control. In this study, a dual‐domain‐engineered anti‐hepatitis B virus (HBV) therapeutic antibody 73‐DY is developed that exhibits significantly improved efficacy regarding both serum and intrahepatic viral clearance. In HBV‐tolerant mice, administration of a single dose of 73‐DY at 2 mg kg^−1^ is sufficient to reduce serum HBsAg by over 3 log_10_ IU mL^−1^ and suppress HBsAg to < 100 IU mL^−1^ for two weeks, demonstrating a dose‐lowering advantage of at least tenfold. Furthermore, 10 mg kg^−1^ of 73‐DY sustainably suppressed serum viral levels to undetectable levels for ≈ 2 weeks. Molecular analyses indicate that the improved efficacy exhibited by 73‐DY is attributable to the synergy between fragment antigen binding (Fab) and fragment crystallizable (Fc) engineering, which conferred sustained viral suppression and robust viral eradication, respectively. Long‐term immunotherapy with reverse chimeric 73‐DY facilitated the restoration of anti‐HBV immune responses. This study provides a foundation for the development of next‐generation antibody‐based CHB therapies.

## Graphical abstract

The dual‐domain engineered anti‐hepatitis B virus antibody 73‐DY undergoes fragment antigen binding (Fab) engineering for pH‐dependent antigen binding, and fragment crystallizable (Fc) engineering for enhancing Fc‐Fcγ receptor interaction. The engineered‐Fc enhances the antibody‐dependent cellular phagocytosis of viral particles, and the engineered‐Fab extends the half‐life of the antibody. Therefore, 73‐DY exhibits higher efficacy in suppressing viremia than the wild‐type antibody, but at a 10‐fold lower dose.

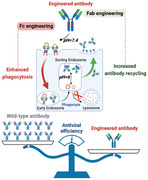

## 1 Introduction

Chronic human hepatitis B virus (HBV) infection is a serious global public health burden.^[^ ^1^, ^2^, ^3^ ^]^ Although effective preventive HBV vaccines are available, new infections and deaths from HBV‐induced liver cirrhosis and hepatocellular carcinoma continue to outpace a cure.^[^ ^4^, ^5^ ^]^ The ultimate endpoint for patients with chronic hepatitis B (CHB) is for hepatitis B surface antigen (HBsAg) to be lost.^[^ ^6^ ^]^ Recent data have shown that the reduction of HBsAg levels can facilitate restoration of the host HBV‐specific immune response and reduce the risk of end‐stage liver disease.^[^ ^7^, ^8^, ^9^, ^10^ ^]^ However, achieving broad effectiveness with first‐line therapeutic agents approved for CHB, such as peginterferon (Peg‐IFN) and nucleoside analogs (NAs), remains elusive.^[^ ^11^ ^]^ Therefore, novel therapeutic strategies are required for improved treatment of patients with chronic HBV infection. Owing to advantages regarding specificity and safety, antibody‐based immunotherapies have demonstrated remarkable potential in treating chronic viral infections.^[^ ^12^, ^13^ ^]^ Recently, monoclonal antibodies (mAbs) targeting HBV have demonstrated rapid and potent clearance of serum HBsAg in patients with CHB and HBV‐carrier mice.^[^ ^14^, ^15^, ^16^ ^]^

However, a high drug dosage or a low viral baseline level is required to achieve ideal therapeutic effects. For example, Lenvervimab (GC1102) can rapidly reduce viral loads in patients with CHB at doses of 80 000—240 000 IU, when HBsAg baseline levels are <1000 IU mL^−1^.^[^ ^15^ ^]^ The HBsAg‐specific antibody G12 can suppress HBsAg levels for over 20 days in hydrodynamic injection‐based HBV mice, but the required dose is 0.5 mg per mouse with HBsAg baseline levels below 500 IU mL^−1^.^[^ ^17^ ^]^ HH‐003 (2H5‐A14), which targets the PreS1 domain, requires a dose of 20 mg kg^−1^ with frequent administration to achieve virological control in human liver‐chimeric FRG mice with baseline HBsAg levels of ≈100 IU mL^−1^.^[^ ^18^ ^]^ E6F6, a murine therapeutic antibody targeting a linear epitope on HBsAg from our previous research, achieved a profound decline in serum HBsAg levels and HBV viremia at a dose of 20 mg kg^−1^.^[^ ^19^ ^]^ Recently, we reported nanobody 125s targeting the C‐terminal end of HBsAg, which exhibited broad‐spectrum in vivo therapeutic activities at a dose of 15 mg kg^−1^.^[^ ^20^ ^]^ High dosages and frequent administration of antibodies have been demonstrated to increase the likelihood of anti‐drug antibody responses and the emergence of drug resistance,^[^ ^21^ ^]^ which is of particular concern in the long‐term treatment of CHB. As a result, there is an urgent need to further develop antibody‐based CHB therapies with a focus on enhancing efficacy and reducing the dosage requirements.

Enhancing antiviral efficacy and reducing the dosage of antiviral therapies through fragment crystallizable (Fc) engineering have demonstrated success in antibody‐based treatments against various viruses. Nirsevimab, an anti‐respiratory syncytial virus prophylactic antibody with an extended half‐life achieved through Fc engineering, provides season‐long protection with a single dose, compared with the five monthly doses required for traditional therapies.^[^ ^22^ ^]^ Anti‐severe acquired respiratory syndrome coronavirus 2 antibodies with enhanced Fc affinity for activating Fcγ receptors (FcγRs) have demonstrated improved in vivo therapeutic activity with the potential for a five‐fold dose reduction.^[^ ^23^ ^]^ Recent studies have attempted to optimize anti‐HBV therapeutic antibodies using Fc engineering. The Fc‐engineered humanized E6F6 exhibited a 2.5‐fold prolonged half‐life in cynomolgus monkeys, but required a dose of 20 mg kg^−1^ for HBsAg clearance in vivo.^[^ ^24^ ^]^ Vir‐3434, which was Fc‐engineered to extend its half‐life and perform T cell‐stimulating functions, effectively reduced HBsAg loads by 1.77 log_10_ IU mL^−1^ at a dose of 75 mg in patients with viremic CHB, whereas serum viremia rapidly rebounded thereafter.^[^ ^25^ ^]^ Despite promising improvements in anti‐HBV antibody efficacy achieved through Fc engineering, dosage reductions have not been achieved. The main obstacle to antibody‐based CHB therapy is the presence of high levels of circulating HBsAg, which can reach up to 4 log_10_ IU mL^−1^, particularly in patients with severe CHB. Therefore, innovative engineering strategies must be developed for next‐generation anti‐HBV therapeutic antibodies.

**Scheme 1:**
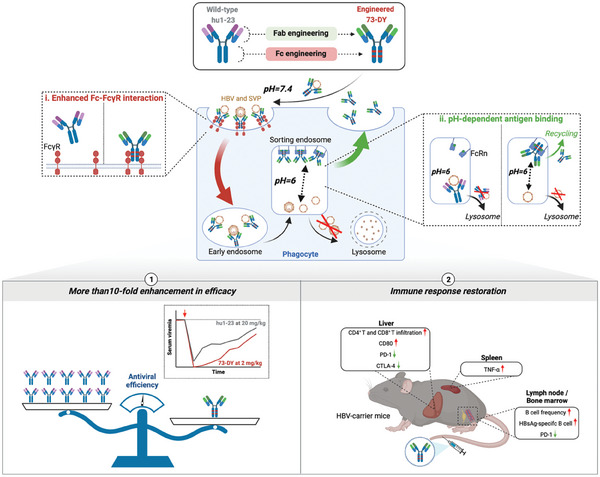
Design of the dual‐domain engineered anti‐HBV therapeutic antibody. Dual‐domain engineering was applied to the wild‐type anti‐HBV antibody hu1‐23. The engineering modification in the Fc domains enhances the FcγR‐dependent antibody‐mediated phagocytosis of viral particles, which translates into enhanced serum and intrahepatic viral clearance. Additionally, Fab engineering confers pH‐dependent antigen‐binding capability to the antibody, thereby enabling the antibody to dissociate from HBV antigens in acidic sorting endosomes, and facilitating antibody recycling. Consequently, the dual‐domain‐engineered anti‐HBV antibody 73‐DY exhibited higher efficacy in viremia suppression compared to the wild‐type antibody, but at a 10‐fold lower dose, indicating its potential to significantly reduce dosing requirements. Moreover, 73‐DY‐based immunotherapy facilitated the reversal of systemic tolerance in HBV carrier mice. This figure was created with *BioRender.com*.

In this study, we developed a novel anti‐HBV antibody, 73‐DY, using dual‐domain engineering, which included fragment antigen binding (Fab) engineering for pH‐dependent HBsAg binding and Fc engineering for enhanced Fc‐dependent effector functions (**Scheme** **1**). In adeno‐associated virus infection‐based (AAV/HBV) mice with high HBsAg titers (reaching 4 log_10_ IU mL^−1^), 73‐DY demonstrated superior therapeutic efficacy in clearing and suppressing virions and subviral particles (SVPs) at a low dose of 2 mg kg^−1^. Immunological analyses suggested that the pH‐dependent HBsAg‐binding property facilitated antibody recycling and prolonged its half‐life and that the enhanced Fc‐FcγR engagement contributed to stronger antibody‐dependent cellular phagocytosis of viral pathogens. Additionally, reverse chimeric 73‐DY‐based immunotherapy achieved long‐term suppression of serum and intrahepatic HBsAg and restored the anti‐HBV T‐cell and B‐cell responses in HBV‐persistent mice. These findings suggest that 73‐DY‐like antibodies may represent promising therapeutic options to treat CHB and provide insights into the therapeutic mechanisms of antibodies against persistent viral infections.

## 2 Results

### 2.1 Fab‐Engineered Anti‐HBV Antibodies with pH‐Dependent HBsAg Binding Prolonged Viral Suppression In Vivo

**Figure 1:**
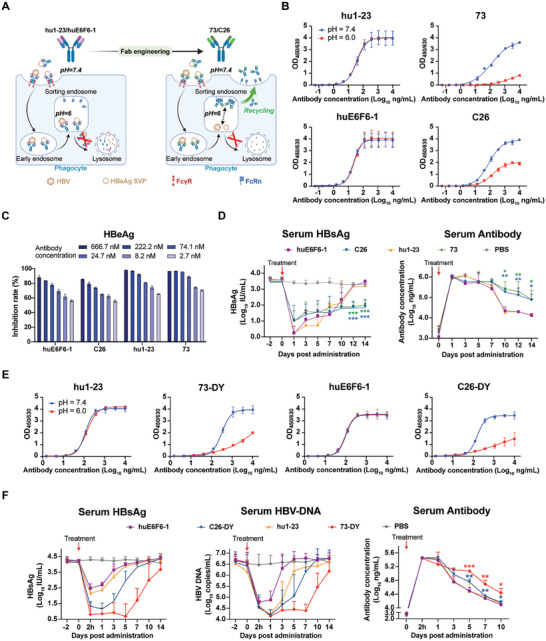
Identification and in vivo therapeutic evaluation of anti‐HBV dual‐domain engineered antibodies. A) Schematic representation of the engineering of anti‐HBV recycling antibody with pH‐dependent HBsAg‐binding property. The recycling antibody can capture HBV and its SVPs at neutral plasma (pH = 7.4) while losing antigen binding in the acidic endosomes (pH = 6.0). The dissociated antibody will be rescued by FcRn and recycled back into the plasma for antigen recapture. This figure was created with *BioRender.com*. B) Binding activities of the antibodies hu1‐23, 73, huE6F6‐1, and C26 against HBsAg under different pH conditions by ELISA analysis (*n* = 3). The data are expressed as the mean ± SD. C) Neutralization of HBV infection in the hNTCP‐expressing cell line by anti‐HBV antibodies (*n* = 3). The levels of HBeAg were used to evaluate the HBV neutralization activity of the antibodies. The data were normalized to the virus infection control and expressed as the mean ± SD. D) Serum HBsAg and antibody levels of AAV/HBV mice after treatment with antibodies or PBS (Control). Each group of mice (*n* = 5) received antibody infusion at a dose of 10 mg kg^−1^. The data are expressed as the mean ± SD. The serum HBsAg and antibody levels of the 73‐ and C26‐treated groups at day 10, 12, and 14 post‐administration were compared to those of the hu1‐23‐ and huE6F6‐1‐treated groups by a two‐sided Student's *t*‐test, respectively (*p < 0.05; **p < 0.01; ***p < 0.001). E) Binding activities of the antibodies hu1‐23, 73‐DY, huE6F6‐1, and C26‐DY against HBsAg under different pH conditions by ELISA analysis (*n* = 3). The data are expressed as the mean ± SD. F) Serum HBsAg, HBV‐DNA, and antibody levels of AAV/HBV mice after treatment with antibody or PBS (Control). Each group of mice (*n* = 4) received antibody infusion at a dose of 5 mg kg^−1^. The data are expressed as the mean ± SD. The serum antibody levels of the 73‐DY‐ and C26‐DY‐treated groups at day 5, 7, and 10 post‐administration were compared to those of the hu1‐23‐ and huE6F6‐1‐treated groups by a two‐sided Student's *t*‐test, respectively (*p < 0.05; **p < 0.01; ***p < 0.001).

We previously obtained two humanized anti‐HBV mAbs, huE6F6‐1 and hu1‐23, with promising HBsAg clearance activities. However, antibody‐based therapy is challenged by high‐level HBsAg in the circulating system and the need for long‐term treatment. Therefore, we sought to improve antibody reutilization by enabling the antibody to capture the viral antigen in neutral plasma (pH 7.4) while dissociating from the antigen in the acidic endosome (pH 6.0), followed by subsequent recycling of the free antibody into the plasma for antigen recapture (**Figure** **1**A). To achieve this, histidine substitution with a *pK*_a_ of pH 6.0, was introduced into the variable regions of huE6F6‐1 and hu1‐23 to confer pH‐dependent HBsAg binding. Two single‐chain variable fragment (scFv) phage libraries were constructed (one each for huE6F6‐1 and hu1‐23), and four rounds of solid‐phase panning were performed (Table S1, Supporting Information). The HBsAg‐binding assays at pH 7.4, and 6.0 showed that 73‐scFv derived from hu1‐23, and C26‐scFv derived from huE6F6‐1, retained their binding affinity at pH 7.4, but significantly lost HBsAg binding at pH 6.0 (Figure S1, Supporting Information). The Fab‐engineered antibodies 73 and C26 with human IgG1 constant regions retained significant pH‐dependent binding to HBsAg (Figure 1B and Table S2, Supporting Information). The Fab‐engineered antibodies exhibited a slight loss of HBsAg‐binding affinity at pH 7.4, which might be attributed to unavoidable amino acid mutations at the antigen‐binding sites (Table S3, Supporting Information). Nevertheless, HBV neutralization assays indicated that the Fab‐engineered antibodies maintained their ability to effectively inhibit HBV infection (Figure 1C).

An AAV/HBV mouse model was used to assess the anti‐HBV therapeutic efficacy of the antibodies in vivo. One day after treatment, significant reductions in the serum HBsAg load were observed in mice that received either wild‐type (hu1‐23 and huE6F6‐1) or Fab‐engineered (73 and C26) antibodies as a single dose of 10 mg kg^−1^ (Figure 1D). Notably, the reduction in serum HBsAg load was more pronounced in mice receiving wild‐type antibodies than in mice receiving Fab‐engineered antibodies by nearly 1 log_10_ IU mL^−1^. The weaker HBsAg clearance by Fab‐engineered antibodies may be due to impaired antigen affinity, as shown in Figure 1B. However, at 12 days post‐administration, HBsAg titers of Fab‐engineered antibody‐treated mice were significantly lower than those of wild‐type antibody‐treated mice by nearly 1.5 log_10_ IU mL^−1^. Furthermore, compared to the serum HBsAg load rebounding to baseline levels by day 12 in the groups treated with hu1‐23 or huE6F6‐1, the serum HBsAg loads in the groups treated with Fab‐engineered antibodies remained below 100 IU mL^−1^ for two weeks and showed a significantly weaker rebound of < 1 log_10_ IU mL^−1^. Interestingly, the improved viral suppression activity of Fab‐engineered antibodies corresponded to their prolonged half‐lives (Figure 1D). From Day 10 onward, the serum concentrations of Fab‐engineered antibodies were significantly higher than those of wild‐type antibodies by ≈1 log_10_ ng mL^−1^. These results suggest that Fab engineering for pH‐dependent HBsAg binding extended the half‐life of the antibody in vivo, thereby prolonging serological viral suppression.

### 2.2 Significant improvement in viremia suppression by further Fc engineering

Although Fab‐engineered antibodies 73 and C26 can prolong viral suppression, an ideal CHB therapy would require further optimization of antibodies that could eradicate HBsAg more effectively. Therefore, we introduced Fc engineering of the double mutations K326D/L328Y, which could improve antibody‐mediated soluble antigen clearance,^[^ ^26^ ^]^ into the Fc regions of 73 and C26 to further engineer the anti‐HBV antibodies, which were denoted as 73‐DY and C26‐DY, respectively. The purity and size of 73‐DY and C26‐DY were confirmed by size exclusion high‐performance liquid chromatography and sodium dodecyl sulfate‐polyacrylamide gel electrophoresis, respectively (Figure S2A,B, Supporting Information). In vitro enzyme‐linked immunosorbent assays and neutralization assays demonstrated that Fc engineering did not affect Fab function, including pH‐dependent HBsAg binding and HBV infection blockade (Figure 1E and Figure S2C, Supporting Information). The morphology of the antibody‐HBsAg immune complexes (ICs) showed that the ICs formed by 73‐DY and C26‐DY remained small and dispersed (Figure S2D, Supporting Information), which may be advantageous for phagocytosis and viral clearance in vivo.^[^ ^19^ ^]^

AAV/HBV mice with initial HBsAg titers of 4.17 ± 0.23 log_10_ IU mL^−1^ and HBV‐DNA titers of 6.63 ± 0.48 log_10_ copies mL^−1^, which are as high as the levels commonly observed in patients with CHB, received a single intravenous infusion of antibodies at a dose of 5 mg kg^−1^. Rapid reductions in the serum HBsAg and HBV DNA loads were observed within 2 hours following administration (Figure 1F). This rapid antiviral response reflects the advantages of passive immunity offered by mAbs. Specifically, 73‐DY potently reduced HBsAg levels by > 3 log_10_ IU mL^−1^ (> 1 log_10_ IU mL^−1^ improvement over hu1‐23) and provided sustained HBsAg suppression. Furthermore, HBV DNA levels in 73‐DY‐treated mice were reduced by over 2 log_10_ copies mL^−1^ and were continuously suppressed at minimal levels for up to 7 days. In contrast, HBV DNA levels in hu1‐23‐treated mice rebounded rapidly and returned to baseline levels by Day 7. Similarly, C26‐DY showed enhanced efficacy in viral clearance, exhibiting over 1 log_10_ IU mL^−1^ enhancement in HBsAg clearance and over 0.5 log_10_ copies mL^−1^ enhancement in HBV‐DNA suppression compared with huE6F6‐1. Analysis of the serum antibody concentration showed that 73‐DY and C26‐DY maintained prolonged antibody half‐lives, which was conferred by Fab engineering, as described above. Among the two engineered antibodies, 73‐DY demonstrated more robust suppression of HBsAg and HBV‐DNA, with subtle changes in alanine aminotransferase levels and routine blood parameters (Figures S3 and S4, Supporting Information). Therefore, 73‐DY was selected for further evaluation of its efficacy and therapeutic potential.

**Figure 2:**
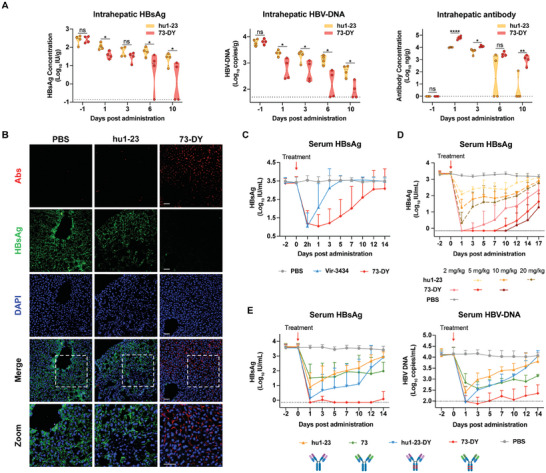
In vivo therapeutic efficacy of 73‐DY. A) Cohorts of AAV/HBV mice (*n* = 4 mice per time point) were injected with hu1‐23 or 73‐DY at a dose of 10 mg kg^−1^ and euthanized at different time points after treatment. The intrahepatic HBsAg, HBV‐DNA, and antibody levels were quantified. *P* values were calculated using a two‐sided Student's *t*‐test (*p < 0.05; **p < 0.01; ****p < 0.0001; “ns” represents not significant). The horizontal dotted lines indicate the lowest detection limits. B) Immunofluorescence staining of HBsAg and antibodies in the liver sections of AAV/HBV mice after 10 mg kg^−1^ of hu1‐23 or 73‐DY infusion or PBS infusion. Assays were performed 6 days post‐treatment. Antibodies (red) were labeled with DyLight 650‐labeled anti‐human IgG, and HBsAg (green) was labeled with DyLight 488‐labeled 129G1‐Fab recognizing the “second loop” linear epitope of HBsAg, which would not be recognized by hu1‐23 or 73‐DY. Representative images from random fields of view in one of the four biologically independent samples. Scale bars of the merge and zoom views: 40 µm. C) Comparison of the therapeutic efficacies of 73‐DY and Vir‐3434 at a dose of 2 mg kg^−1^ in AAV/HBV mice (*n* = 5 mice per group). Serum HBsAg was quantified and the data are expressed as the mean ± SD. D) Serum HBsAg levels of mice (*n* = 5 mice per group) treated with 73‐DY and hu1‐23 at indicated doses (2, 5, and 10 mg kg^−1^ for 73‐DY; 5, 10, and 20 mg kg^−1^ for hu1‐23). The data are expressed as the mean ± SD. The horizontal dotted line indicates the lowest detection limit. E) Serum HBsAg and HBV‐DNA profiles of AAV/HBV mice receiving hu1‐23, hu1‐23‐DY, 73, 73‐DY, or PBS (Control). Each group of mice (*n* = 5) received antibody infusion at a dose of 10 mg kg^−1^. The data are expressed as the mean ± SD. The horizontal dotted lines indicate the lowest detection limits.

To evaluate the clearance of intrahepatic HBsAg during antibody treatment, cohorts of AAV/HBV mice received either hu1‐23 or 73‐DY, and the levels of intrahepatic HBsAg, HBV‐DNA, and antibodies were quantified at different time points. The results revealed that intrahepatic viral levels in 73‐DY‐treated mice were consistently lower than those in hu1‐23‐treated mice (**Figure** **2**A). Notably, by post‐treatment Day 10, 73‐DY eliminated the intrahepatic HBsAg and HBV‐DNA to undetectable levels in two out of four mice, while hu1‐23 treatment failed to reduce intrahepatic viral loads to the same extent. Additionally, a strong correlation was observed between intrahepatic viral and antibody levels. In mice treated with 73‐DY, intrahepatic antibody levels were persistently higher than those in hu1‐23‐treated mice (Figure 2A). By post‐treatment Day 10, intrahepatic hu1‐23 levels were nearly undetectable, whereas intrahepatic 73‐DY levels remained above 3 Log_10_ ng g^−1^. Immunofluorescence analysis of the liver tissues collected on Day 6 post‐infusion further indicated that 73‐DY treatment resulted in lower intrahepatic HBsAg levels and higher intrahepatic antibody levels (Figure 2B). These findings suggest that 73‐DY exhibits systematically improved viral clearance and suppression, including effects against serum and intrahepatic HBV antigens.

In comparison with the clinically tested antibody Vir‐3434, which has proven efficacy in reducing HBsAg levels in chronically infected patients in a phase 1 trial (NCT04423393), 73‐DY demonstrated comparable HBsAg reduction. However, 73‐DY exhibited a more sustained suppression of viremia than that of Vir‐3434, with serum HBsAg suppression lasting for 14 and 5 days, respectively (Figure 2C). To investigate the dose‐effect relationship, hu1‐23 and 73‐DY were administered at specific dose gradients (2, 5, and 10 mg kg^−1^ for 73‐DY; 5, 10, and 20 mg kg^−1^ for hu1‐23). The results revealed that 73‐DY significantly enhanced HBsAg clearance compared to hu1‐23 at the same doses (Figure 2D). Specifically, HBsAg levels in mice treated with 2 mg kg^−1^ 73‐DY were reduced by over 3 log_10_ IU mL^−1^ and suppressed to below 100 IU mL^−1^ for two weeks, which were consistently lower than those in mice treated with 20 mg kg^−1^ hu1‐23. These findings suggest that 73‐DY can achieve better HBsAg seroclearance at a 10‐fold lower dose than hu1‐23. To further investigate the impact of Fab and Fc engineering on the therapeutic effects of 73‐DY, antibodies with different modifications, including the wild‐type antibody hu1‐23, Fab‐engineered antibody 73, Fc‐engineered antibody hu1‐23‐DY, and dual‐domain‐engineered antibody 73‐DY, were evaluated in vivo (Figure 2E). As expected, Fab‐engineered antibody 73 exhibited weaker HBsAg and HBV‐DNA reductions, but significantly prolonged viral suppression compared to hu1‐23. In contrast, the Fc‐engineered antibody hu1‐23‐DY, which was designed by applying the same Fc engineering to hu1‐23, exhibited much stronger viral reduction than hu1‐23. However, viral levels in mice treated with hu1‐23‐DY rebounded rapidly and returned to baseline levels on Day 14. Finally, the dual‐domain‐engineered 73‐DY demonstrated similarly enhanced viral eradication as hu1‐23‐DY and prolonged viral suppression as 73. Specifically, the infusion of 10 mg kg^−1^ 73‐DY profoundly suppressed serum HBsAg and HBV DNA loads to undetectable levels for ≈ 2 weeks. These data suggest that the synergy between Fab and Fc engineering accounted for the improved efficacy of 73‐DY. Specifically, Fab engineering contributed to sustained viremia suppression, whereas Fc engineering conferred intense and rapid clearance of viral particles.

### 2.3 The Therapeutic Activity of 73‐DY is Associated with Increased Antibody Recycling and Enhanced Antibody‐Dependent Cellular Phagocytosis of Viral Pathogens

**Figure 3:**
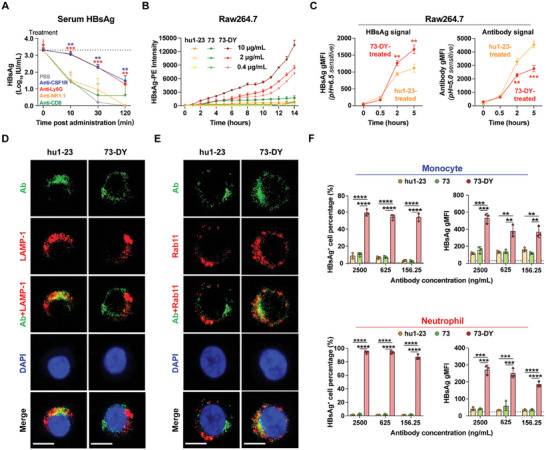
73‐DY achieved increased antibody recycling through Fab engineering and mediated enhanced cellular phagocytosis of viral pathogens through Fc engineering. A) The roles that different effector immune cells played in 73‐DY‐mediated viral clearance in AAV/HBV mice (*n* = 5 mice per group). Depletion of monocytes/macrophages (anti‐CSF1R), neutrophils (anti‐Ly6G), NK cells (anti‐NK 1.1), or CD8^+^ T cells (anti‐CD8α) was performed 1 day before 73‐DY infusion. The serum HBsAg levels were quantified and are expressed as the mean ± SD. The *P* values were calculated by comparing to the values from the PBS‐treated group using a two‐sided Student's *t*‐test (**p < 0.01; ***p < 0.001). The dotted line indicates the average level of HBsAg before treatment. B) Dynamic monitoring of antibody‐mediated phagocytosis of HBsAg by Raw264.7 cells with an IncuCyte SX5 Live‐Cell Analysis Instrument (*n* = 3). HBsAg was labeled with a pH 6.5‐sensitive dye to emit fluorescence once phagocytosed. The fluorescence intensity was calculated using IncuCyte evaluation software, and the data are expressed as the mean ± SD. C) In vitro antibody‐mediated HBsAg phagocytosis in Raw264.7 cells (*n* = 3). HBsAg was labeled with a pH 6.5‐sensitive dye as described above, and antibodies (hu1‐23 and 73‐DY) were labeled with a pH 5.0‐sensitive dye that only emits deep red fluorescence in lysosomes. Flow cytometry was used to detect the intracellular fluorescence intensity and the gMFI of HBsAg or antibody was calculated respectively. The data are expressed as the mean ± SD. The *P* values were calculated by comparing to the values from the hu1‐23‐treated group using a two‐sided Student's *t*‐test (**p < 0.01; ***p < 0.001). (D‐E) Confocal microscopy images of Raw264.7 cells incubated with hu1‐23‐ or 73‐DY‐HBsAg immune complexes to show distribution of antibodies and lysosomes or recycling endosomes. hu1‐23 or 73‐DY was pre‐incubated with recombinant HBsAg for 60 min and then added to Raw264.7 cells for a further 60 min. Cells were washed, fixed, and permeabilized. Antibodies (green) were labeled with DyLight 488‐labeled mouse anti‐human IgG (H+L) secondary antibody. Lysosomes or recycling endosomes (red) were labeled with rabbit anti‐mouse lysosome‐associated membrane protein‐1 (LAMP‐1) D) or rabbit anti‐mouse Rab11 E), respectively, followed by DyLight 568‐labeled donkey anti‐rabbit IgG. The co‐localization between antibodies and lysosomes or recycling endosomes is shown in yellow (overlap of green and red). Representative images from random fields of view in one of the three biologically independent samples. Scale bar: 10 µm. F) hu1‐23, 73, and 73‐DY induced in vitro phagocytosis of HBsAg in primary murine monocytes and neutrophils (*n* = 3). Cells were evaluated by flow cytometric analysis, and the percentage of HBsAg^+^ cells and the HBsAg gMFI in these phagocytes were calculated. The horizontal dotted lines indicate the percentages of HBsAg^+^ cells and the HBsAg gMFI for spontaneous HBsAg phagocytosis in the absence of antibody treatment. The data are expressed as the mean ± SD. The *P* values were calculated using a two‐sided Student's *t*‐test (**p < 0.01; ***p < 0.001; ****p < 0.0001).

We conducted a detailed investigation of the antiviral mechanism of 73‐DY following its favorable efficacy results. To identify specific effector cell types associated with the antiviral effects of 73‐DY, we performed selective cell depletion, including depletion of monocytes/macrophages (anti‐CSF1R), neutrophils (anti‐Ly6G), natural killer (NK) cells (anti‐NK1.1) and CD8+ T cells (anti‐CD8α) before administering 73‐DY. Serum HBsAg levels showed that the depletion of phagocytes, including monocytes/macrophages and neutrophils, significantly reduced the HBsAg clearance effect of 73‐DY (**Figure** **3**A). These results suggest that 73‐DY likely achieves potent antiviral clearance by enhancing antibody‐dependent cellular phagocytosis of viral particles. To determine the efficiency of antibody‐mediated cellular phagocytosis, we introduced a real‐time imaging system to periodically measure the fluorescence intensity of phagocytized HBsAg, which was labeled with a pH 6.5‐sensitive dye that fluoresced only upon entering acidic endosomes, in the monocyte/macrophage cell line Raw264.7. The data showed that 73‐DY induced faster and more potent cellular phagocytosis of HBsAg than hu1‐23 and 73 in a dose‐dependent manner (Figure 3B). In addition, we used Raw264.7 cells to assess 73‐DY‐mediated antigen phagocytosis and antibody recycling. In this assay, HBsAg was labeled with a pH 6.5‐sensitive dye, whereas the antibodies were labeled with a pH 5.0‐sensitive dye that fluoresced only in lysosomes. Periodic measurements of internalized HBsAg and antibodies showed that compared to hu1‐23, 73‐DY exerted stronger HBsAg phagocytosis efficiency and more efficient escape from the lysosome (Figure 3C). These observations were further supported by immunofluorescence analysis of Raw264.7 cells incubated with hu1‐23‐ or 73‐DY‐HBsAg ICs. Specifically, after a 60‐minute incubation period, the majority of internalized hu1‐23 localized within lysosomes (labeled with anti‐lysosome associated membrane protein‐1 (LAMP‐1)), whereas 73‐DY was distributed away from lysosomes (Figure 3D). Moreover, the distribution of antibodies and recycling endosomes (labeled with anti‐Rab11), which facilitate antibody recycling back to the cell surface, was examined in Raw264.7 cells. Notably, 73‐DY was predominantly distributed in recycling endosomes, while no significant co‐localization between hu1‐23 and recycling endosomes was observed (Figure 3E). These data suggest that Fab engineering endowed 73‐DY with increased lysosomal escape and subsequent recycling, leading to an extended antibody half‐life. Additionally, Fc engineering of 73‐DY enhanced the antibody‐dependent cellular phagocytosis (ADCP) of HBV antigens.

We proceeded to investigate the involvement of FcRn, which was highly expressed in recycling endosomes and closely related to IgG recycling, in the recycling of 73‐DY.^[^ ^27^ ^]^ Consistent with the immunofluorescence results demonstrating the co‐localization of 73‐DY with recycling endosomes, the internalized 73‐DY within Raw264.7 cells exhibited a significantly higher level of co‐localization with FcRn compared to hu1‐23 (Figure S5, Supporting Information). Additionally, we pre‐treated Raw264.7 cells with either wild‐type Fc (Fc‐WT) or an Fc variant called Fc‐MST‐HN (efgartigimod), which is known to effectively block Fc‐FcRn binding, in the 73‐DY‐mediated HBsAg phagocytosis models (Figure S6A, Supporting Information).^[^ ^28^ ^]^ Immediately after the incubation of 73‐DY‐HBsAg ICs with the cells (no chase), we observed higher levels of 73‐DY accumulation in cells pre‐treated with Fc‐MST‐HN compared with those in cells pretreated with Fc‐WT. Moreover, during the chase period, a significantly larger amount of 73‐DY was recycled out of the cells pre‐treated with Fc‐WT compared with that in Fc‐MST‐HN‐treated cells (Figure S6B, Supporting Information). Furthermore, the half‐life of 73‐DY was substantially shortened in FcRn knockout mice (Figure S7, Supporting Information). Collectively, these data emphasize the crucial role of FcRn in the recycling of 73‐DY and the maintenance of its in vivo homeostasis.

Furthermore, we used several *ex vivo* phagocytosis models, including those established using primary murine monocytes and neutrophils, to validate these findings. Flow cytometry results revealed that 73‐DY treatment significantly enhanced ADCP compared with hu1‐23 and 73 in a dose‐dependent manner, as evidenced by an increased percentage of HBsAg phagocytic cells (HBsAg^+^ cell percentage), and improved efficiency of HBsAg phagocytosis (HBsAg gMFI) (Figure 3F). The antibody recycling efficiency of 73‐DY and its predecessors was also analyzed in monocytes and neutrophils. As shown in Figure S6C (Supporting Information), both 73‐DY and 73, engineered for pH‐dependent antigen‐binding properties in the Fab regions, demonstrated higher levels of antibody recycling, with >40% and 20% of the antibodies being recycled out of the monocytes and neutrophils during the chase period, respectively. However, the recycling percentages of hu1‐23 were only 18.1% in monocytes and 9.8% in neutrophils. Additionally, early endosomes and lysosomes were extracted from monocytes and neutrophils of AAV/HBV mice treated with 73‐DY or hu1‐23, and the antibody levels were characterized in these organelles using western blotting analysis. The concentrations of 73‐DY in early endosomes were significantly higher than those of hu1‐23, while the opposite trend was observed in lysosomes (Figure S8, Supporting Information). These findings validate the enhanced HBsAg phagocytosis and antibody recycling activities mediated by 73‐DY in monocytes and neutrophils.

Subsequently, we sought to determine whether 73‐DY could also improve viral phagocytosis in the peripheral blood of mice containing heterogeneous mixtures. To achieve this, fresh peripheral blood from AAV/HBV mice with an average HBsAg titer of 1634.12 IU mL^−1^ was incubated with HBsAg and tested antibodies in an *ex vivo* phagocytosis assay. The results revealed that compared to 73, 73‐DY induced significantly enhanced phagocytosis of HBsAg in neutrophils and monocytes from the peripheral blood (Figure S9, Supporting Information). Moreover, we conducted another ex vivo phagocytosis assay by incubating antibody‐antigen ICs with fresh non‐parenchymal cells isolated from the liver tissues of AAV/HBV mice, which contain large numbers of phagocytes with distinct phenotypes and play a crucial role in clearing circulating and intrahepatic HBV antigens. Similarly, 73‐DY significantly enhanced HBsAg phagocytosis in major types of intrahepatic phagocytes, including Kupffer cells (KCs), monocyte‐derived macrophages, and neutrophils (Figure S10, Supporting Information). In addition, an immunofluorescence assay of mouse livers collected on Day 6 after antibody treatment revealed that 73‐DY not only had a higher intrahepatic antibody concentration but also showed significantly increased co‐localization with F4/80^+^ and Ly6C/Ly6G^+^ cells, which are typical markers of monocytes, macrophages, and neutrophils, revealing increased interactions between 73‐DY and the intrahepatic phagocytes (Figure S11, Supporting Information). Overall, these results suggest that 73‐DY can enhance the cellular phagocytosis of HBV antigens by various phagocytes from different tissues, including the peripheral blood and liver.

### 2.4 Enhancement of the Fc‐FcγR Interaction Contributes to the 73‐DY‐Induced Improvement in ADCP

**Figure 4:**
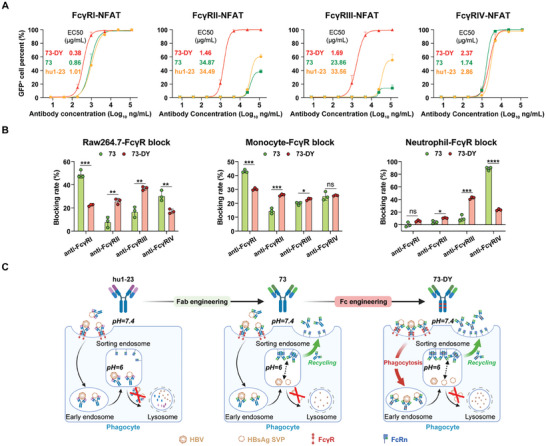
Increased Fc‐FcγR interactions conferred the enhanced ADCP activity of 73‐DY. A) Identification of antibody‐mediated signaling activation with four genetically modified 2B4 cell lines expressing specific FcγRs with an inducible GFP luciferase reporter gene (*n* = 3). Fluorescence was analyzed by confocal microscopy, and the percentage of GFP^+^ cells was calculated by Columbus Analysis system. The data are expressed as the mean ± SD, and a nonlinear regression best‐fit curve was generated for each dataset. EC50 values were calculated by GraphPad Prism (v.9.0). B) Comparison of the reductions in 73‐ and 73‐DY‐mediated ADCP after complete blocking of specific FcγRs (*n* = 3). The blocking rate was calculated with the following formula: [(gMFI of 73‐DY‐mediated phagocytosis without FcγR blocking – gMFI of 73‐DY‐mediated phagocytosis with indicated FcγR blocking) / (gMFI of 73‐DY‐mediated phagocytosis without FcγR blocking – gMFI of spontaneous phagocytosis without 73‐DY treatment)] × 100%. The data are expressed as the mean ± SD. *P* values were calculated using a two‐sided Student's *t*‐test (*p < 0.05; **p < 0.01; “ns” represents not significant). C) Schematic representation of the dual‐domain engineered anti‐HBV antibody, 73‐DY. This antibody exhibits an enhanced capacity to promote phagocytosis of HBV antigens by increasing its interaction with FcγRs. Upon the internalization of antibody‐antigen immune complexes into sorting endosomes with a pH of 6, the antibody dissociates from the antigen and undergoes recycling back to the plasma via binding with FcRn. Meanwhile, the antigen is subjected to degradation within lysosomes. This figure was created with BioRender.com.

The Fc regions of an antibody can interact with FcγRs on the surface of effector cells to trigger a series of effector functions, including phagocytosis of opsonized pathogens.^[^ ^29^ ^]^ Therefore, to evaluate the molecular mechanism of 73‐DY‐mediated increased phagocytosis of HBV antigens, we characterized the affinity of the antibodies for four classes of mouse FcγRs using surface plasmon resonance (SPR) analysis (**Table** **1** and Figure S12, Supporting Information). The results showed that 73‐DY exhibited significantly enhanced affinity for FcγRII and FcγRIII compared to hu1‐23, as evidenced by lower affinity constants (*K*_D_) of 30.86‐ and 19.42‐fold, respectively. A 4.87‐fold increased affinity was also observed between 73‐DY and the high‐affinity receptor FcγRI. To further verify the FcγR‐dependent signaling activation mediated by Fc‐FcγR engagement, we introduced four genetically engineered 2B4 reporter cell lines that express specific classes of FcγRs on the cell surface and rely on FcγR cross–linking to trigger downstream immunoreceptor tyrosine‐based activation motif (ITAM; for FcγRI, FcγRIII, and FcγRIV)‐ or immunoreceptor tyrosine‐based inhibitory motif (ITIM; for FcγRII)‐mediated nuclear factor of activated T cells (NFAT)‐GFP reporter activation.^[^ ^30^ ^]^ Consistent with the SPR data, the ICs formed with 73‐DY and HBsAg significantly enhanced the activation of FcγRII‐ and FcγIII‐dependent signaling, as evidenced by 23.62‐ and 19.86‐fold decreases in the EC50 values compared to those of hu1‐23‐based ICs, respectively. A 2.7‐fold increase in 73‐DY‐induced activation of FcγRI‐dependent signaling was also observed (**Figure** **4**A). These data indicated that 73‐DY had greatly improved affinity for FcγRII and FcγRIII, with slightly enhanced affinity for FcγRI, and that the enhanced Fc‐FcγR interaction was associated with increased FcγR‐dependent signaling activation.

**Table 1:** Characterization of the affinities of hu1‐23, 73, and 73‐DY for murine FcγRs using BIAcore analysis.

Antibody	FcγRI		FcγRII		FcγRIII		FcγRIV	
	*K*_D_ [M]	Fold	*K*_D_ [M]	Fold	*K*_D_ [M]	Fold	*K*_D_ [M]	Fold
hu1‐23	5.41E‐08	1.00	3.00E‐07	1.00	2.33E‐07	1.00	4.18E‐08	1.00
73	4.52E‐08	1.20	3.79E‐07	0.79	1.29E‐07	1.81	4.26E‐08	0.98
73‐DY	1.11E‐08	4.87	9.72E‐09	30.86	1.20E‐08	19.42	5.43E‐08	0.77

The fold change was calculated in affinity compared with that of hu1‐23.

Furthermore, we introduced specific FcγR‐blocking antibodies before the ADCP assays, which have been shown to effectively and specifically block corresponding Fc‐FcγR binding.^[^ ^31^, ^32^ ^]^ The results showed that 73‐DY‐mediated HBsAg phagocytosis was effectively reduced with specific FcγR blocking in a dose‐dependent manner in various phagocytosis models (Figure S13, Supporting Information). Notably, due to the distinct FcγR expression profile on neutrophils, which predominantly express FcγRIII and FcγRIV,^[^ ^33^ ^]^ only blockade of FcγRIII and FcγRIV produced a significant reduction in ADCP by neutrophils. We also observed that combinations of blocking antibodies achieved stronger blocking effects than individual blocking antibodies (Figure S14A, Supporting Information). Therefore, we conducted a complete blockade of specific classes of FcγR to compare the reductions in 73‐ and 73‐DY‐mediated phagocytosis of HBsAg in different models. As expected, the reduction of ADCP activity in Raw264.7 cells and primary monocytes upon completely blocking FcγRII and FcγRIII were substantially more robust with 73‐DY treatment than those with 73 treatment, indicating that the enhanced phagocytosis of HBsAg in Raw264.7 cells and monocytes mediated by 73‐DY was attributed to the increased engagement with FcγRII and FcγRIII (Figure 4B and Figure S14B, Supporting Information). Similarly, in primary neutrophils, the contribution of FcγRIII‐dependent phagocytosis was significantly increased with 73‐DY treatment compared to that of 73 treatment. These findings demonstrated the molecular mechanism underlying 73‐DY‐mediated improved ADCP activity, in which the enhanced phagocytosis of HBsAg in monocytes/macrophages relied on increased interaction with FcγRII and FcγRIII, while the enhanced phagocytosis in neutrophils was due to increased interaction with FcγRIII.

In summary, we proposed a model (Figure 4C) that summarizes a dual‐domain antibody engineering strategy that enhances antibody‐mediated HBV clearance and suppression. Specifically, Fc‐engineering in 73‐DY increases Fc‐FcγR engagement, resulting in enhanced cellular phagocytosis of viral particles, thereby improving viral seroclearance. Additionally, Fab‐engineering for pH‐dependent HBsAg binding enables the dissociation of 73‐DY and viral antigens in acidic endosomes, allowing the antibody to evade lysosomal degradation and be recycled into the plasma with FcRn for repeated virus capture. Consequently, 73‐DY has an extended half‐life, leading to prolonged suppression of viremia.

### 2.5 73‐DY‐based Immunotherapy Enhances Humoral and Cellular Immune Responses in HBV‐Tolerant Mice

**Figure 5:**
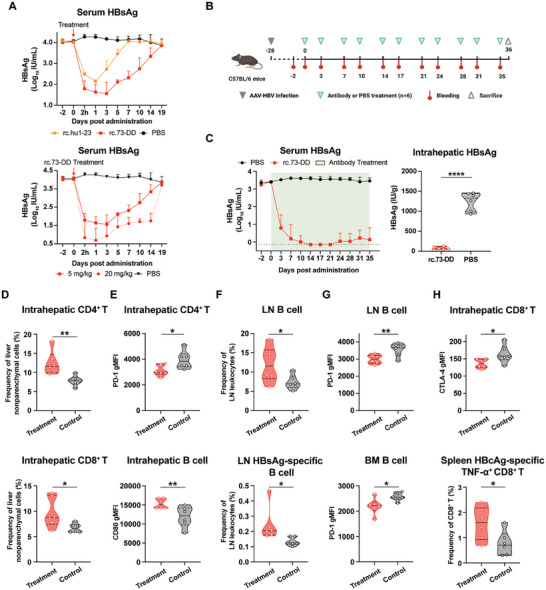
The restoration of immune responses in AAV/HBV mice by reverse chimeric 73‐DY‐based immunotherapy. A) Serum HBsAg levels of AAV/HBV mice after antibody or PBS (Control) infusion at the dose of 5 mg kg^−1^ (*n* = 5 mice per group). The data are expressed as the mean ± SD. B) Diagram of the experimental procedure. Six mice were used in each group. At each time point of antibody or PBS injection, blood samples were collected in advance. This figure was created with BioRender.com. C) Serum HBsAg levels during the whole course and intrahepatic HBsAg quantification at the end of the treatment period. The data of serum HBsAg are expressed as the mean ± SD. *P* values in the intrahepatic HBsAg data were calculated using a two‐sided Student's *t*‐test (****p < 0.0001). The horizontal dotted line indicates the lowest detection limit. (D‐H) Comparison of immune responses between the rc.73‐DD‐treated group and the control group by flow cytometric analysis (*n* = 6). Data were statistically analyzed by a two‐sided Student's *t*‐test (*p < 0.05; **p < 0.01). D) Frequencies of T cells in liver nonparenchymal cells. E) PD‐1 expression profiles of intrahepatic CD4^+^ T cells (the upper row) and CD80 expression profiles of intrahepatic B cells (the lower row). F) B‐cell and HBsAg‐specific B‐cell frequencies in the lymph nodes. G) PD‐1 expression profiles of B cells from the lymph nodes and bone marrow. H) Profiles of CD8^+^ T‐cell responses. CTLA‐4 expression profiles of intrahepatic CD8^+^ T cells (the upper row) and the frequencies of HBcAg‐specific TNF‐α secreting CD8^+^ T cells in the spleens (the lower row).

Persistent exposure to HBV antigens is crucial for HBV‐specific humoral and cellular immune tolerance, and sustained control of the HBsAg load has been proven effective in restoring anti‐HBV immune responses.^[^ ^7^, ^34^, ^35^, ^36^ ^]^ Therefore, we aimed to investigate the immune‐modulating effects of 73‐DY‐like antibodies in AAV/HBV mice. Since repeated administration of 73‐DY as human IgG1 induced a certain degree of mouse anti‐human antibody responses in mice, we generated a reverse chimeric version of 73‐DY (rc.73‐DD) by switching the constant regions of 73‐DY to murine IgG1 subtype and introducing Fc mutations for enhanced affinity to FcγRII and FcγRIII (Table S4, Figures S15 and S16, Supporting Information). In vivo treatment revealed that rc.73‐DD could achieve stronger HBsAg seroclearance than the reverse chimeric version of hu1‐23 (rc.hu1‐23), which contains variable regions of hu1‐23 and wild‐type murine IgG1 constant regions, and that HBsAg seroclearance mediated by rc.73‐DD was dose‐dependent (**Figure** **5**A). Therefore, a therapeutic strategy aimed at persistently suppressing serum and intrahepatic viral levels using rc.73‐DD or PBS (control) was developed in AAV/HBV mice (*n* = 6 mice per group) (Figure 5B). Liver and immune tissues collected on day 36 post‐treatment were subjected to flow cytometric analysis of immune cell responses (Figure S17, Supporting Information). The results showed that rc.73‐DD significantly suppressed serum HBsAg loads from 1753.34 IU mL^−1^ to < 3.16 (10^0.5^) IU mL^−1^ for four weeks, and the levels of intrahepatic HBsAg in mice treated with rc.73‐DD were also significantly reduced by > 1000 IU g^−1^ compared to those in the control group at the end of the treatment period (Figure 5C).

To evaluate the immune responses elicited by rc.73‐DD‐mediated immunotherapy, we first analyzed the infiltration of immune cells into the liver. Compared to the control group, the rc.73‐DD‐treated group showed significant increases in CD4^+^ and CD8^+^ T cells in the liver (Figure 5D). Additionally, an increased frequency of B cells, although not statistically significant, was observed in the livers of rc.73‐DD‐treated mice (Figure S18, Supporting Information). These results suggest that rc.73‐DD‐mediated therapy has the potential to reverse humoral and cellular immune tolerances. Therefore, we first explored the changes in other humoral immune response profiles. Significantly reduced expression of the co‐inhibitory receptor programmed cell death protein‐1 (PD‐1) was observed in intrahepatic CD4^+^ T cells treated with rc.73‐DD (Figure 5E, upper row), which is known to participate in HBV‐induced T cell tolerance with programmed cell death 1 ligand 1 (PD‐L1).^[^ ^37^, ^38^, ^39^ ^]^ Moreover, we identified remarkably increased expression of the costimulatory receptor CD80 in intrahepatic B cells, indicating potential B cell activation in the liver (Figure 5E, lower row). In peripheral immune tissues, significant increases in the number of B cells and HBsAg‐specific B cells were observed in the lymph nodes (LNs) of mice receiving rc.73‐DD treatment (Figure 5F). Furthermore, we identified a less exhausted phenotype, defined as downregulated PD‐1 expression, in LN B cells (Figure 5G, upper row). B cells with significantly lower levels of PD‐1 were also found in the bone marrow (BM), which might be attributed to the homing of LN B cells (Figure 5G, lower row). These data suggest that immunotherapy based on reverse chimeric 73‐DY suppresses viremia and intrahepatic viral levels and facilitates significant restoration of the systemic antiviral humoral immune response.

CD8^+^ T cell exhaustion is a crucial mechanism underlying cellular immune tolerance in patients with CHB and is characterized by reduced proliferative capacity, high expression of co‐inhibitory receptors, and decreased cytokine secretion.^[^ ^39^ ^]^ After identifying the increased CD8^+^ T‐cell infiltration in the liver after rc.73‐DD treatment, we further discovered reduced expression of the co‐inhibitory receptor cytotoxic T lymphocyte‐associated protein‐4 (CTLA‐4) in intrahepatic CD8^+^ T cells (Figure 5H, upper row), which has been reported to be overexpressed and to mediate the premature attrition of CD8^+^ T cells in CHB patients.^[^ ^40^ ^]^ Furthermore, we examined the cytokine secretion profiles and observed a significant increase in the level of hepatitis B core antigen (HBcAg)‐specific tumor necrosis factor‐α (TNF‐α)‐secreting CD8^+^ T cells in the spleens of rc.73‐DD‐treated mice (Figure 5H, the lower row). TNF‐α has been shown to be involved in multiple anti‐HBV mechanisms.^[^ ^41^ ^]^ Together, these results suggest that, in addition to humoral immune tolerance, long‐term virological control with reverse chimeric 73‐DY can effectively reverse multiple exhausted phenotypes of anti‐HBV cellular immunity.

After identifying the activation of adaptive immune responses following long‐term rc.73‐DD therapy, we further investigated whether this treatment strategy would lead to sustained HBsAg clearance in vivo (Figure S19, Supporting Information). As expected, the antibody infusions effectively resulted in a substantial elimination of serum HBsAg. Following the cessation of antibody treatment, HBsAg titers returned to baseline within 2 months in four out of six mice. However, in the remaining two mice, HBsAg levels continued to be suppressed (<10 IU mL^−1^) for >2 months despite stopping antibody therapy (Figure S19A, Supporting Information). Notably, these two mice exhibited consistently high levels of serum anti‐HBs, which persisted for 70 days following treatment cessation, suggesting the in vivo production of spontaneous anti‐HBs responses (Figure S19B, Supporting Information). These findings underscore the capability of long‐term viremia suppression with reverse chimeric 73‐DY in inducing sustained HBsAg clearance and facilitating the restoration of antiviral immune responses.

## 3 Discussion

Although antibody‐based therapies have demonstrated promising antiviral efficacy against CHB in clinical trials, high‐dose administration is required, highlighting the need for next‐generation anti‐HBV therapeutic antibodies with significantly improved efficacy and reduced dosing requirements. In this study, we combined the pH‐dependent HBsAg‐binding Fab engineering and the FcγR‐specific Fc engineering to construct dual‐domain engineered antibodies. This strategy greatly improves the in vivo antiviral efficacy and produces a more than 10‐fold dose‐lowering effect, thereby providing a cost‐effective, easy‐to‐administer (e.g., subcutaneous route), and safer option for the long‐term treatment of CHB. It is widely accepted that achieving a functional cure for CHB requires combination therapies, and monoclonal antibodies combined with other drugs have shown promising efficacy in suppressing viremia and restoring antiviral immune responses.^[^ ^17^, ^42^, ^43^ ^]^ Therefore, we suggest that 73‐DY‐like therapeutic antibodies, which can serve as a more efficient HBsAg scavenger, may be preferred for combination therapies aimed at increasing the HBsAg loss rate and improving the clinical management of CHB.

The most widely used approach to extend the antibody half‐life is FcRn‐specific Fc engineering, which increases IgG affinity for FcRn at acidic pH.^[^ ^44^ ^]^ FcRn‐specific Fc engineering of anti‐HBV therapeutic antibodies has exhibited limited improvement in efficacy.^[^ ^24^ ^]^ Fab engineering has also proven effective in extending antibody half‐life.^[^ ^45^, ^46^ ^]^ Our study is the first to apply Fab engineering for pH‐dependent antigen binding to HBsAg‐specific therapeutic antibodies. The pH‐dependent binding property allows an antibody to capture the HBV antigen in neutral plasma, but dissociates from the antigen within the acidic endosome, enabling it to be recycled to the plasma to recapture new antigens. Our findings revealed that although there was a slight decrease in antigen‐binding affinity, which resulted in a weaker serum HBsAg eradication capacity, Fab engineering provided a significantly prolonged antibody half‐life, leading to a remarkably sustained suppression of viremia, which compensated for the loss of HBsAg eradication in long‐term observation. Moreover, we enhanced the ADCP function of pH‐dependent HBsAg‐binding antibodies through Fc engineering, further improving antibody‐mediated viral clearance. The critical role of ADCP in the clearance of infectious pathogens, including HBV and its SVPs,^[^ ^19^ ^]^ has been widely demonstrated.^[^ ^47^, ^48^, ^49^ ^]^ Molecular analyses revealed that increased IgG affinities for FcγRII and FcγRIII directly contribute to 73‐DY‐mediated enhanced ADCP. Importantly, the combination of Fab and Fc engineering synergistically enhanced viral clearance and suppression.

In this study, we compared the therapeutic efficacy of 73‐DY and the clinically tested Vir‐3434 in an HBV carrier mouse model. Vir‐3434 underwent Fc engineering alone, whereas 73‐DY underwent dual‐domain engineering. The rapid and potent HBsAg seroclearance provided by 73‐DY was comparable to that provided by Vir‐3434 at an equivalent dose. However, viremia rebounded rapidly in Vir‐3434‐treated mice, which is consistent with the findings in viremic CHB patients who received Vir‐3434 treatment in a phase 1 trial. In contrast, 73‐DY provided a more profound suppression of viral loads, indicating the benefit of additional Fab engineering for pH‐dependent HBsAg binding to 73‐DY to prolong antibody‐mediated viral suppression. Notably, the rapid HBsAg rebound in Vir‐3434‐treated mice may be due to the divergence of FcRn between humans and mice, as Vir‐3434 relies on increased binding to human FcRn to extend the antibody half‐life. Learning from the Vir‐3434 efficacy in reducing HBsAg by 1.77 log_10_ IU mL^−1^ at a dose of 75 mg per CHB patient,^[^ ^25^ ^]^ we speculate that 73‐DY‐like antibodies can be superior candidates for providing potent HBV clearance and suppression at low doses in CHB therapy.

Clinical studies have shown that drug therapies that achieve long‐term suppression of HBsAg facilitate the functional recovery of HBV‐specific T‐cell‐ and B‐cell responses.^[^ ^7^, ^50^ ^]^ In this study, we investigated the immune‐modulating effects of 73‐DY‐like antibodies in acquired HBV‐tolerant mice. Reverse chimeric 73‐DY‐based immunotherapy suppresses viremia for a month and restores the intrahepatic immune environment, characterized by increased frequencies and less exhausted phenotypes of CD4^+^ and CD8^+^ T cells. Notably, restoration of intrahepatic T cells is usually more efficient than that of peripheral T cells.^[^ ^51^ ^]^ The analysis of humoral immune responses detected multiple B cell activation signals, including an increase in virus‐specific B cells, upregulation of CD80, and upregulation of HBcAg‐specific cytotoxic T lymphocytes from the spleen. There is compelling evidence that HBcAg induces stronger T‐cell responses than envelope antigens,^[^ ^52^ ^]^ and that cytokines secreted by HBV‐specific T‐cells have direct antiviral effects and immunomodulatory functions.^[^ ^41^, ^53^ ^]^ Together, our findings demonstrate the immunomodulatory benefits of 73‐DY‐based immunotherapy, which promotes the reversal of adaptive immunotolerance in the liver and peripheral immune tissues of AAV/HBV mice. We hypothesized that prolonged treatment might further improve antiviral immune responses.

In conclusion, we present a functionally optimized anti‐HBV therapeutic antibody that incorporates Fab engineering for pH‐dependent antigen binding and Fc engineering for enhanced ADCP function. The dual‐domain‐engineered antibody 73‐DY demonstrated at least a 10‐fold increase in potency for viremia suppression in vivo, together with the benefits of restoring virus‐specific immune responses. Anti‐HBV therapeutic antibodies with pH‐dependent HBsAg binding properties and enhanced Fc affinities for specific human FcγRs may overcome the existing high‐dose requirements and promote antibody‐based CHB therapies. We propose that this study provides a general strategy for developing antibody‐based therapies against chronic viral infections that are associated with high pathogen burdens.

## 4 Experimental Section

### Mice

C57BL/6 mice were purchased from SLAC, CN. To construct an adaptive HBV‐tolerant model, an AAV/HBV plasmid (serotype adw, Packgene, CN) that contained 1.3 copies of the HBV genome was hydrodynamically injected into the tail vein of C57BL/6 mice at 5 × 10^10^ GC per dose in PBS.^[^ ^54^ ^]^ All mice were maintained under specific pathogen‐free conditions in the Laboratory Animal Center of Xiamen University. The experiments were conducted with the approval of the Institutional Animal Care and Use Committee at Xiamen University (XMULAC20150016) and in accordance with the Guide for the Care and Use of Laboratory Animals.

### Screening and Construction of pH‐Dependent HBsAg Binding Antibodies

Cloning primers with degenerate oligonucleotides were used to introduce histidine mutations randomly in the complementarity‐determining regions (CDRs) of humanized monkey‐derived hu1‐23^[^ ^55^ ^]^ and mouse‐derived huE6F6‐1,^[^ ^56^ ^]^ and recombinant PCGMT‐expression vectors expressing scFvs were transformed into ER2738 electrocompetent cells (Lucigen, US) and selected with ampicillin (Sangon Biotech, CN).^[^ ^57^ ^]^ The helper phage M13KO7 (NEB, US) was used to infect ER2738 cells, and phage‐scFv primary libraries for hu1‐23 and huE6F6‐1 with capacities of 6.7 × 10^7^ and 1.15 × 10^8^, respectively, were constructed. Library biopanning was carried out against a recombinant HBsAg protein (CHO cell‐derived, Wantai Biological Pharmacy Enterprise Co., Ltd., CN) coated on 96‐well ELISA plates, as shown in Figure S1A (Supporting Information). Four rounds of panning were performed, and clones with a stronger binding affinity for HBsAg at pH 7.4 than at pH 6.0 were defined as pH‐dependent positive (Figure S1B, Supporting Information). The genes of the variable heavy chain (vH) and light chain (vL) of the positive clones were sequenced. The heavy chain and light chain variable regions of selected scFv clones were subcloned into pTT5 expression vectors (YouBio, CN) containing the human and murine IgG1 heavy chain and kappa chain constant regions, respectively.^[^ ^58^ ^]^

### Antibody Production

K326D/L328Y and S239D/A327D mutations were introduced into the Fc regions of human IgG1 and murine IgG1, respectively, by site‐directed mutagenesis to generate the DY‐Fc variant and DD‐Fc variant. Expi293F cells (Gibco, Thermo Fisher Scientific, US) were transiently cotransfected with the heavy chain and kappa chain expression plasmids at a 1:1 ratio using polyethylenimine (PEI; Polyscience, Chicago) and cultured for 7 days. Antibodies were purified from culture supernatants using protein A affinity chromatography (General Electric Company, Pittsburgh, PA) and identified by sodium dodecyl sulfate‐polyacrylamide gel electrophoresis (SDS‒PAGE).

### pH‐Dependent HBsAg Binding ELISA

Recombinant HBsAg protein (2 µg mL^−1^) was coated onto ELISA 96‐well plates. A series of 3‐fold dilutions for each tested antibody was added to the reaction wells and incubated for 60 min at 37 °C. After washing, 100 µL of pH 6.0 or pH 7.4 PBS was added to the wells and incubated for 30 min at 37 °C. pH 6.0 or pH 7.4 PBST, respectively, was subsequently applied for washing, followed by a reaction with a horseradish peroxidase (HRP)‐conjugated anti‐human polyclonal antibody (pAb) (Thermo Scientific, Rockford, USA). Finally, o‐phenyl‐diamine‐2HCl was added as a substrate for 15 min. The reaction was stopped by 2 m H_2_SO_4_, and the absorbance was measured at 450 nm, with automatic subtraction of the reference absorbance at 630 nm.

### In Vitro HBV Neutralization Test

HepaRG cells were inoculated in 24‐well plates four days in advance. HBV derived from the culture supernatant of HepaAD38 cells and diluted antibodies were incubated for 1 h at 37 °C and then added to HepaRG cells with 4% (w/v) PEG8000 and 2% dimethyl sulfoxide (DMSO). After overnight incubation, the HepaRG cells were gently washed three times with fresh medium containing 2% DMSO and cultured for an additional 6 days. On the 7th day after infection, hepatitis B envelope antigen (HBeAg) in the culture medium was quantified using a chemiluminescence kit purchased from Wantai Biological Pharmacy Enterprise Co., Ltd.

### Virological Assays to Detect HBV Markers

Serum and intrahepatic HBsAg titers were measured using an HBsAg chemiluminescent quantitation kit purchased from Wantai Biological Pharmacy Enterprise Co., Ltd. HBV‐DNA in serum was extracted using the Universal Genomic DNA Extraction Kit (GenMagBio, CN). Real‐time PCR was conducted to measure the level of HBV‐DNA in the serum using TransStart Probe qPCR SuperMix (TransGen Biotech, CN).

### Detection of Antibody Concentration and Anti‐HBs

To determine the human IgG‐based antibody concentrations in serum and hepatic tissue samples, 96‐well ELISA plates were precoated with an anti‐human IgG antibody (Fab specific; Sigma‒Aldrich, USA) and appropriately blocked. Serum or liver lysate samples were diluted and added to the reaction wells. Antibodies were detected with an HRP‐conjugated anti‐human kappa light chain secondary antibody (Invitrogen, USA), and a chromogenic substrate and stop solution was subsequently used. The antibody concentrations were quantified by using human IgG from serum (Sigma‒Aldrich, USA) with known concentrations as standards.

For the detection of serum ant‐HBs levels, recombinant HBsAg protein (2 µg mL^−1^) was coated onto ELISA 96‐well plates. Serum samples were diluted 50‐fold and added to pre‐coated HBsAg. The presence of mouse anti‐HBs was detected using HRP‐conjugated goat anti‐mouse IgG (H+L) secondary antibody (ab6789, Abcam, UK). The following steps were performed according to the manufacturer's protocols.

### In Vivo Cell Depletion

Monocytes/macrophages, neutrophils, NK cells, or CD8^+^ T cells were depleted in AAV/HBV mice by administration of anti‐CSF1R, anti‐Ly6G, anti‐NK1.1 or anti‐CD8 monoclonal antibodies, respectively. One day before 73‐DY infusion, AAV/HBV mice were intraperitoneally administered 500 µg of anti‐mouse CSF1R monoclonal antibody (Clone: AFS98, Catalog: BE0213), anti‐mouse Ly6G monoclonal antibody (Clone: 1A8, Catalog: BE0075‐1), anti‐mouse NK1.1 monoclonal antibody (Clone: PK136, Catalog: BE0036), anti‐mouse CD8α monoclonal antibody (Clone: 2.43, Catalog: BE0061), or PBS.

### In Vitro and Ex Vivo Phagocytosis Assays

In the phagocytosis assays, HBsAg was labeled with a pH 6.5‐sensitive dye (pHrodo iFL Red Microscale Protein Labeling Kits, Invitrogen, USA). Raw264.7 cells were purchased from ATCC (USA). Murine monocytes and neutrophils were isolated from the spleen and BM with the EasySep Mouse Monocyte Isolation Kit (STEMCELL, Canada) and EasySep Mouse Neutrophil Enrichment Kit (STEMCELL, Canada), respectively. Peripheral blood was harvested from mice and red blood cells were lysed on ice using red blood cell lysis buffer (Solarbio, CN). Peripheral blood leukocytes were processed for surface labeling with appropriate antibodies according to the manufacturer's instructions. Liver nonparenchymal cells were isolated from the liver as described previously.^[^ ^19^ ^]^ Antibodies to be tested were separately diluted and incubated with 400 ng mL^−1^ HBsAg at a 1:1 ratio for 1 h at 37 °C. After the incubation, the mixture was added to the cells incubated at 37 °C for 2 h. After washing, the cells were subjected to flow cytometric analysis according to the manufacturer's instructions. To dynamically evaluate phagocytosis, an Incucyte SX5 Live‐Cell Analysis Instrument (Sartorius, Germany) was used to monitor the red fluorescence signal from internalized HBsAg every hour, and the fluorescence intensity was calculated using Incucyte evaluation software (Sartorius, Germany). To evaluate the antibody‐mediated HBsAg phagocytosis efficiency and antibody recycling, Antibody was labeled with a pH 5.0‐sensitive dye (pHrodo Deep Red Antibody Labeling Kits, Invitrogen, USA). 2 µg mL^−1^ antibody and 2 µg mL^−1^ HBsAg were co‐incubated at a 1:1 ratio for 1 h at 37 °C and added to Raw264.7 cells. The Raw264.7 cells were subjected to flow cytometric analysis at indicated time points.

### Western Blotting Assays

Monocytes and neutrophils were isolated from the peripheral blood of mice as described above. Early endosomes and lysosomes were isolated using Minute Endosome Isolation and Cell Fractionation Kit and Minute Endosome Isolation and Cell Fractionation Kit according to the manufacturer's protocols, respectively (Invent Biotechnologies, USA). The isolated organelles were lysed using RIPA lysis buffer (50 mM Tris,150 mM NaCl, 1% Triton X‐100, 1% sodium deoxycholate, and 0.1% SDS). SDS‐polyacrylamide gel electrophoresis (SDS‐PAGE) was operated with 10 µg of proteins per well at a voltage of 100 V for 90 min. The proteins were transferred to a polyvinylidene difluoride membrane using wet transfer mode (Biorad, USA) operated at 260 mA for 50 min. Membranes were incubated in primary antibodies overnight at 4 °C and then washed with TBS containing 0.05% Tween‐20 for five times each for 5 min. Membranes were then incubated with HRP‐coupled secondary antibodies for 1 h at room temperature followed by washing five times. The detection of proteins was performed using enhanced chemiluminescence (ECL) and the results were analyzed and quantified by ImageJ.

### FcγR‐Specific Blocking in Phagocytosis Assays

Mouse FcγRs were blocked by an anti‐mouse FcγRI monoclonal antibody (Clone: 290322, Catalog: MAB20741), anti‐mouse FcγRII monoclonal antibody (Clone: 190907, Catalog: MAB14601), anti‐mouse FcγRIII monoclonal antibody (Clone: 275003, Catalog: MAB19601) or anti‐mouse FcγRIV monoclonal antibody (Clone: 9E9, Catalog: 149502). The specific blocking activity of these antibodies was demonstrated in published studies^[^ ^31^, ^32^ ^]^ and was described in the corresponding product datasheets (https://www.rndsystems.com/). In this study, Raw264.7 cells, primary monocytes, and neutrophils were preincubated with diluted FcγR‐blocking antibodies for 30 min at 37 °C. Then, the tested antibody–HBsAg mixture was added to the cells and co‐incubated for 1 h at 37 °C. The cells were washed with PBS and subjected to flow cytometric analysis.

### Immunofluorescence Microscopy

Liver samples were taken from mice on day 6 after antibody injection. A piece of each liver sample was fixed with 4% paraformaldehyde for 8 h and then infiltrated with 20% sucrose for 30 min and 30% sucrose overnight for dehydration. The samples were embedded in an OCT‐embedding medium (SAKURA Tissue‐Tek O.C.T. Compound, SAKURA, Japan). Cryosections (15 µm) were cut on a cryostat (Leica CM1950, Leica, Germany). After washing with PBS, the cryosections were treated with 0.3% Triton X‐100 for 10 min and blocked in 5% BSA for 1 h. The cryosections were incubated overnight at 4 °C with the following antibodies: anti‐F4/80 (Clone: A3‐1, ThermoFisher, USA) and anti‐Ly6C/Ly6G (NIMP‐R14, Novus Biologicals, USA). Then, anti‐F4/80 and anti‐Ly6C/Ly6G were detected with an Alexa Fluor 568‐conjugated goat anti‐rat IgG secondary antibody (ab175476, Abcam, UK) at room temperature for 1 h. HBsAg and 73‐DY/hu1‐23 were detected with 129G1‐Cab‐Dylight 488 and Goat anti‐Human IgG Fc Cross‐Adsorbed Secondary Antibody, DyLight 650, respectively. Images were acquired on a confocal microscope (TCS SP8 X, Leica, Germany).

### SPR

SPR experiments were performed on a BIAcore 8K (BIAcore, GE Healthcare, USA) at 25 °C in HBS‐EP+ buffer (Cytiva, USA). Mouse FcγRs were purchased from Sino Biological (Beijing, CN) with a 6×His tag fused to the C‐terminus of the FcγRs. Briefly, antibodies with hIgG‐Fc were immobilized on a Series S Protein A sensor chip (GE Healthcare, USA) at a 300‐response unit (RU) density. Antibodies with mIgG‐Fc were captured on a CM5 sensor chip (GE Healthcare, USA) on which anti‐mouse antibodies had been immobilized. Serial dilutions of recombinant FcγRs were injected into the flow cells at 30 µL min^−1^, at concentrations ranging from 400 to 3.125 nM (1:2 successive dilutions). The association time was 90 s, followed by a 120‐s dissociation step. At the end of each cycle, the sensor surface was regenerated by a glycine HCl buffer (10 mM, pH 1.7; 50 µL min^−1^, 30 s). Background binding to blank immobilized flow cells was subtracted, and *K*_D_ values were calculated using BIAcore8K evaluation software (GE Healthcare, USA) with the 1:1 Langmuir binding model.

### FcγR‐Dependent Signaling Activation in the NFAT‐GFP Reporter Cell Line

Genetically engineered T hybridoma 2B4 cells were designed to express mouse FcγRI, FcγRII, FcγRIII, or FcγRIV on the surface.^[^ ^30^ ^]^ Upon Fc‐induced cross–linking of FcγRs, the intracellular ITAM or ITIM is phosphorylated, inducing NFAT activation and subsequent GFP fluorescence. In this study, antibodies to be tested were separately diluted and incubated with 200 ng mL^−1^ HBsAg for 1 h at 37 °C. After the incubation, the mixture was added to the appropriate FcγR‐NFAT‐GFP cells at 100 µL per well and incubated at 37 °C for 48 h. The cells were evaluated on a high‐content imaging system (Opera Phenix, Perkin Elmer), and fluorescence images were acquired. Image data were analyzed with the Columbus system (version 2.5.0), and the percentage of green fluorescent (GFP^+^) cells was calculated.

### Flow Cytometric Analysis

For phenotypic analysis of leukocytes, antibodies specific for 4‐1BB (Clone: 17B5, Catalog: 106103), CD11b (Clone: M1/70, Catalog: 101215), CD11c (Clone: N418, Catalog: 117343), CD19 (Clone: 1D3/CD19, Catalog: 152403), CD28 (Clone: 37.51, Catalog: 102106), CD4 (Clone: GK1.5, Catalog: 100434), CD40 (Clone: 3/23, Catalog: 124611), CD40L (Clone: MR1, Catalog: 106509), CD80 (Clone: 16‐10A1, Catalog: 104708), CD8α (Clone: 53–6.7, Catalog: 100711), CTLA‐4 (Clone: BNI3, Catalog: 369603), ICOS (Clone: C398.4A, Catalog: 313533), LAG‐3 (Clone: C9B7W, Catalog: 125227), LY6G (Clone: 1A8, Catalog: 127639), NK1.1 (Clone: PK136, Catalog: 108749), OX40 (Clone: OX‐86, Catalog: 119419), PD‐1 (Clone: 29F.1A12, Catalog: 135225), TIGIT (Clone: 1G9, Catalog: 142108), and TIM3 (Clone: RMT3‐23, Catalog: 119721) were purchased from BioLegend. The antibody specific for CD45 (Clone: 30‐F11, Catalog: 564279) was purchased from BD. The antibody specific for F4/80 (Clone: REA126, Catalog: 130‐116‐499) was purchased from Miltenyi Biotec. Leukocytes isolated from the peripheral blood (PB), ILNs, spleen, BM, or liver were processed for surface labeling with appropriate antibodies according to the manufacturer's instructions. The Zombie Aqua Fixable Viability Kit (BioLegend) was used to determine cell viability. All the cells were analyzed by flow cytometry using an LSR Fortessa X‐20 (BD Biosciences). The data were analyzed using FlowJo software (FlowJo, Ashland, OR, USA). For antiviral cytokine analysis, antibodies specific for CD3 (Clone: 17A2, Catalog: 100222), CD4 (Clone: GK1.5, Catalog: 100434), CD8α (Clone: 53–6.7, Catalog: 100753), IFN‐γ (Clone: XMG1.2, Catalog: 505808), and TNF‐α (Clone: MP6‐XT22, Catalog: 506308) were purchased from BioLegend. Leukocytes isolated from the spleen were stimulated with synthetic peptides for 24 h. BD GolgiPlug Protein Transport Inhibitor (containing Brefeldin A) was added 4 h prior to surface staining. After surface staining, the cells were resuspended in Fixation/Permeabilization solution (BD Cytofix/Cytoperm kit; BD), and intracellular cytokine staining was performed according to the manufacturer's protocol.

### Statistical Analysis

The in vitro neutralization of HBV infection was normalized to the virus infection control. GraphPad Prism (v.9.0), FlowJo (v.10.6.2), and ImageJ (v.1.8.0) were used for data visualization, statistical analysis, and figure production. All the quantitative data were presented as mean ± standard deviation (SD) with at least three independent experiments. Detailed sample sizes (*n*) were labeled in the figures or figure legends, and the sample size for each statistical analysis was at least three. The two‐tailed Student's t‐test was used to determine the statistical significance between the two groups. The statistical significance was indicated as “ns” (not significant), *p < 0.05, **p < 0.01, ***p < 0.001, and ****p < 0.0001, 95% confidence level.

## Conflict of Interest

The author declares no conflicts of interest.

## Author Contributions

Y.J., X.C., and X.Y. contributed equally to this work. Y.J., C.W., T.Z., and W.L. were involved in conceptualization. Data curation was done by Y.J., X.C., X.Y., T.X., C.Y., W.N., X.L. The formal analysis was conducted by Y.J., X.C., X.Y., Y.W. N.X., W.L., T.Z. and Y.C. helped in funding acquisition. The methodology involved Y.J., G.W., X.X, and X.L. Resource allocation was overseen by Q.Y., Y.C., G.W., and Y.C. N.X. and W.L. provided valuable supervision. The writing team had Y.J. and X.C. for the original draft and received critical input and refinement from C.S., C.L., T.Z., W.L., and N.X. during the review and editing phase.

## Supporting information

Supporting Information

## Data Availability

The data that support the findings of this study are available from the corresponding author upon reasonable request.

## References

[1] S. Asrani , H. Devarbhavi , J. Eaton , P. Kamath , J Hepatol 2019, 70, 151. 30266282 10.1016/j.jhep.2018.09.014

[2] L. Tang , E. Covert , E. Wilson , S. Kottilil , JAMA, J. Am. Med. Assoc. 2018, 319, 1802. 10.1001/jama.2018.3795 29715359

[3] J. Wang , W. Wu , T. Huang , M. Wong , K. Kwak , K. Ozato , C. Hung , R. Roden , J. Virol. 2018, 92. 10.1128/JVI.00572-18 PMC6052307 29743371

[4] World Health Organization . Hepatitis B. 2023 July 18 [cited 2023 October]; Available from: https://www.who.int/en/news‐room/fact‐sheets/detail/hepatitis‐b.

[5] W. Jeng , G. Papatheodoridis , A. Lok , Lancet 2023, 401, 1039. 36774930 10.1016/S0140-6736(22)01468-4

[6] A. Vittal , M. Ghany , Clin. Liver Dis. 2019, 23, 417. 31266617 10.1016/j.cld.2019.04.008 PMC9616205

[7] F. Rinker , C. Zimmer , C. Höner Zu Siederdissen , M. Manns , A. Kraft , H. Wedemeyer , N. Björkström , M. Cornberg , J. Hepatol. 2018, 69, 584. 29758333 10.1016/j.jhep.2018.05.004

[8] H. Wang , H. Luo , X. Wan , X. Fu , Q. Mao , X. Xiang , Y. Zhou , W. He , J. Zhang , Y. Guo , W. Tan , G. Deng , J. Hepatol. 2020, 72, 45. 31499130 10.1016/j.jhep.2019.08.024

[9] T. Yip , G. Wong , H. Chan , Y. Tse , K. Lam , G. Lui , V. Wong , J. Hepatol. 2019, 70, 361. 30367899 10.1016/j.jhep.2018.10.014

[10] C. Song , J. Zhu , Z. Ge , C. Yu , T. Tian , H. Wang , J. Han , H. Shen , J. Dai , J. Lu , Z. Hu , Clin. Gastroenterol. Hepatol. 2019, 17, 1204. 30114488 10.1016/j.cgh.2018.08.019

[11] N. Terrault , A. Lok , B. Mcmahon , K. Chang , J. Hwang , M. Jonas , R. Brown , N. Bzowej , J. Wong , Clin. Liver Dis. 2018, 12, 33. 10.1002/cld.728 PMC6385899 30988907

[12] S. Bournazos , A. Gazumyan , M. Seaman , M. Nussenzweig , J. Ravetch , Cell 2016, 165, 1609. 27315478 10.1016/j.cell.2016.04.050 PMC4970321

[13] J. Scheid , J. Horwitz , Y. Bar‐On , E. Kreider , C. Lu , J. Lorenzi , A. Feldmann , M. Braunschweig , L. Nogueira , T. Oliveira , I. Shimeliovich , R. Patel , L. Burke , Y. Cohen , S. Hadrigan , A. Settler , M. Witmer‐Pack , A. West , B. Juelg , T. Keler , T. Hawthorne , B. Zingman , R. Gulick , N. Pfeifer , G. Learn , M. Seaman , P. Bjorkman , F. Klein , S. Schlesinger , B. Walker , et al., Nature 2016, 535, 556. 27338952 10.1038/nature18929 PMC5034582

[14] A. Neumann , I. Feldberg , R. Eren , S. Dagan , Hepatology 2003, 38, 709a.

[15] H. Lee , J. Park , T. Hong , M. Park , S. Ahn , Clin. Gastroenterol. Hepatol. 2020, 18, 3043. 31589980 10.1016/j.cgh.2019.09.038

[16] D. Zhu , L. Liu , D. Yang , S. Fu , Y. Bian , Z. Sun , J. He , L. Su , L. Zhang , H. Peng , Y. Fu , J. Immunol. 2016, 196, 3079. 26936879 10.4049/jimmunol.1502061 PMC4824405

[17] B. Shi , Y. Wu , C. Wang , X. Li , F. Yu , B. Wang , Z. Yang , J. Li , M. Liang , Y. Wen , T. Ying , Z. Yuan , EBioMedicine 2019, 49, 247. 31680000 10.1016/j.ebiom.2019.10.043 PMC6945269

[18] D. Li , W. He , X. Liu , S. Zheng , Y. Qi , H. Li , F. Mao , J. Liu , Y. Sun , L. Pan , K. Du , K. Ye , W. Li , J. Sui , Elife 2017, 6. 10.7554/eLife.26738 PMC5614562 28949917

[19] T. Zhang , Q. Yuan , J. Zhao , Y. Zhang , L. Yuan , Y. Lan , Y. Lo , C. Sun , C. Wu , J. Zhang , Y. Zhang , J. Cao , X. Guo , X. Liu , X. Mo , W. Luo , T. Cheng , Y. Chen , M. Tao , J. Shih , Q. Zhao , J. Zhang , P. Chen , Y. Yuan , N. Xia , Gut 2016, 65, 658. 26423112 10.1136/gutjnl-2014-308964

[20] Y. Wang , Y. Mei , Z. Ao , Y. Chen , Y. Jiang , X. Chen , R. Qi , B. Fu , J. Tang , M. Fang , M. You , T. Zhang , Q. Yuan , W. Luo , N. Xia , Antiviral Res. 2022, 199, 105265. 35183645 10.1016/j.antiviral.2022.105265

[21] S. Inzaule , T. Rinke de Wit , R. Hamers , N. Engl. J. Med. 2018, 378, 873. 10.1056/NEJMc1716089 29504387

[22] Q. Zhu , J. McLellan , N. Kallewaard , N. Ulbrandt , S. Palaszynski , J. Zhang , B. Moldt , A. Khan , C. Svabek , J. McAuliffe , D. Wrapp , N. Patel , K. Cook , B. Richter , P. Ryan , A. Yuan , J. Suzich , Sci. Transl. Med. 2017, 9. 10.1126/scitranslmed.aaj1928 28469033

[23] R. Yamin , A. Jones , H. Hoffmann , A. Schäfer , K. Kao , R. Francis , T. Sheahan , R. Baric , C. Rice , J. Ravetch , S. Bournazos , Nature 2021, 465, 599. 10.1038/s41586-021-04017-w PMC9038156 34547765

[24] C. Kang , L. Xia , Y. Chen , T. Zhang , Y. Wang , B. Zhou , M. You , Q. Yuan , C. Tzeng , Z. An , W. Luo , N. Xia , Protein Cell 2018, 9, 130. 28677103 10.1007/s13238-017-0438-y PMC5777975

[25] K. Agarwal , M. Yuen , H. Wedemeyer , D. Cloutier , L. Shen , A. Arizpe , S. Gupta , M. Fanget , L. Seu , A. Cathcart , A. Lau , C. Hwang , E. Gane , Hepatology 2022, 76, S303.

[26] Y. Iwayanagi , T. Igawa , A. Maeda , K. Haraya , N. Wada , N. Shibahara , K. Ohmine , T. Nambu , G. Nakamura , F. Mimoto , H. Katada , S. Ito , T. Tachibana , K. Jishage , K. Hattori , J. Immunol. 2015, 195, 3198. 26320252 10.4049/jimmunol.1401470 PMC4574519

[27] G. Vidarsson , A. Stemerding , N. Stapleton , S. Spliethoff , H. Janssen , F. Rebers , M. De Haas , J. Van De Winkel , Blood 2006, 108, 3573. 16849638 10.1182/blood-2006-05-024539

[28] P. Ulrichts , A. Guglietta , T. Dreier , T. Van Bragt , V. Hanssens , E. Hofman , B. Vankerckhoven , P. Verheesen , N. Ongenae , V. Lykhopiy , F. Enriquez , J. Cho , R. Ober , E. Ward , H. De Haard , N. Leupin , J. Clin. Invest. 2018, 128, 4372. 30040076 10.1172/JCI97911 PMC6159959

[29] X. Wang , M. Mathieu , R. Brezski , Protein Cell 2018, 9, 63. 28986820 10.1007/s13238-017-0473-8 PMC5777978

[30] F. Saito , K. Hirayasu , T. Satoh , C. Wang , J. Lusingu , T. Arimori , K. Shida , N. Palacpac , S. Itagaki , S. Iwanaga , E. Takashima , T. Tsuboi , M. Kohyama , T. Suenaga , M. Colonna , J. Takagi , T. Lavstsen , T. Horii , H. Arase , Nature 2017, 552, 101. 29186116 10.1038/nature24994 PMC5748893

[31] P. Zhang , G. Tu , J. Wei , P. Santiago , L. Larrabee , S. Liao‐Chan , T. Mistry , M. Chu , T. Sai , K. Lindquist , H. Long , J. Chaparro‐Riggers , S. Salek‐Ardakani , Y. Yeung , Cell Rep. 2019, 27, 3117. 31189099 10.1016/j.celrep.2019.05.027

[32] M. Otten , G. Van Der Bij , S. Verbeek , F. Nimmerjahn , J. Ravetch , R. Beelen , J. Van De Winkel , M. Van Egmond , J. Immunol. 2008, 181, 6829. 18981101 10.4049/jimmunol.181.10.6829

[33] C. Kerntke , F. Nimmerjahn , M. Biburger , Front. Immunol. 2020, 11. 10.3389/fimmu.2020.00118 PMC7013094 32117269

[34] A. Burton , L. Pallett , L. Mccoy , K. Suveizdyte , O. Amin , L. Swadling , E. Alberts , B. Davidson , P. Kennedy , U. Gill , C. Mauri , P. Blair , N. Pelletier , M. Maini , J. Clin. Invest. 2018, 128, 4588. 30091725 10.1172/JCI121960 PMC6159997

[35] A. Bertoletti , C. Ferrari , J Hepatol 2016, 64, S71. 27084039 10.1016/j.jhep.2016.01.026

[36] C. Boni , D. Laccabue , P. Lampertico , T. Giuberti , M. Viganò , S. Schivazappa , A. Alfieri , M. Pesci , G. Gaeta , G. Brancaccio , M. Colombo , G. Missale , C. Ferrari , Gastroenterology 2012, 143, 963. 22796241 10.1053/j.gastro.2012.07.014

[37] Y. Dong , X. Li , L. Zhang , Q. Zhu , C. Chen , J. Bao , Y. Chen , BMC Immunol. 2019, 20, 27. 31390978 10.1186/s12865-019-0309-9 PMC6686459

[38] L. Salimzadeh , N. Le Bert , C. Dutertre , U. Gill , E. Newell , C. Frey , M. Hung , N. Novikov , S. Fletcher , P. Kennedy , A. Bertoletti , J. Clin. Invest. 2018, 128, 4573. 30084841 10.1172/JCI121957 PMC6159957

[39] B. Ye , X. Liu , X. Li , H. Kong , L. Tian , Y. Chen , Cell Death Dis. 2015, 6. 10.1038/cddis.2015.42 PMC4385920 25789969

[40] A. Schurich , P. Khanna , A. Lopes , K. Han , D. Peppa , L. Micco , G. Nebbia , P. Kennedy , A. Geretti , G. Dusheiko , M. Maini , Hepatology 2011, 53, 1494. 21360567 10.1002/hep.24249

[41] Y. Xia , D. Stadler , J. Lucifora , F. Reisinger , D. Webb , M. Hösel , T. Michler , K. Wisskirchen , X. Cheng , K. Zhang , W. Chou , J. Wettengel , A. Malo , F. Bohne , D. Hoffmann , F. Eyer , R. Thimme , C. Falk , W. Thasler , M. Heikenwalder , U. Protzer , Gastroenterology 2016, 150, 194. 26416327 10.1053/j.gastro.2015.09.026

[42] G. Fanning , F. Zoulim , J. Hou , A. Bertoletti , Nat Rev Drug Discov 2019, 18, 827. 31455905 10.1038/s41573-019-0037-0

[43] E. Gane , A. Jucov , M. Dobryanska , K. Yoon , T. Lim , A. Arizpe , D. Cloutier , L. Shen , S. Gupta , A. Lau , C. Hwang , Y. Lim , Hepatology 2022, 76, S18.

[44] W. Dall'acqua , P. Kiener , H. Wu , J. Biol. Chem. 2006, 281, 23514. 16793771 10.1074/jbc.M604292200

[45] A. Traboulsee , B. Greenberg , J. Bennett , L. Szczechowski , E. Fox , S. Shkrobot , T. Yamamura , Y. Terada , Y. Kawata , P. Wright , A. Gianella‐Borradori , H. Garren , B. Weinshenker , Lancet Neurol. 2020, 19, 402. 32333898 10.1016/S1474-4422(20)30078-8 PMC7935419

[46] T. Igawa , H. Tsunoda , T. Tachibana , A. Maeda , F. Mimoto , C. Moriyama , M. Nanami , Y. Sekimori , Y. Nabuchi , Y. Aso , K. Hattori , Protein Eng Des Sel 2010, 23, 385. 20159773 10.1093/protein/gzq009

[47] M. Ackerman , A. Dugast , E. Mcandrew , S. Tsoukas , A. Licht , D. Irvine , G. Alter , J. Virol. 2013, 87, 5468. 23468489 10.1128/JVI.03403-12 PMC3648186

[48] D. Dilillo , P. Palese , P. Wilson , J. Ravetch , J. Clin. Invest. 2016, 126, 605. 26731473 10.1172/JCI84428 PMC4731186

[49] C. Nelson , T. Huffman , J. Jenks , E. Cisneros De La Rosa , G. Xie , N. Vandergrift , R. Pass , J. Pollara , S. Permar , Proc Natl Acad Sci USA 2018, 115, 6267. 29712861 10.1073/pnas.1800177115 PMC6004431

[50] M. Bazinet , V. Pântea , G. Placinta , I. Moscalu , V. Cebotarescu , L. Cojuhari , P. Jimbei , L. Iarovoi , V. Smesnoi , T. Musteata , A. Jucov , U. Dittmer , A. Krawczyk , A. Vaillant , Gastroenterology 2020, 158, 2180. 32147484 10.1053/j.gastro.2020.02.058

[51] P. Fisicaro , C. Valdatta , M. Massari , E. Loggi , E. Biasini , L. Sacchelli , M. Cavallo , E. Silini , P. Andreone , G. Missale , C. Ferrari , Gastroenterology 2010, 138, 682. 19800335 10.1053/j.gastro.2009.09.052

[52] G. Lau , D. Suri , R. Liang , E. Rigopoulou , M. Thomas , I. Mullerova , A. Nanji , S. Yuen , R. Williams , N. Naoumov , Gastroenterology 2002, 122, 614. 11874993 10.1053/gast.2002.31887

[53] L. Guidotti , T. Ishikawa , M. Hobbs , B. Matzke , R. Schreiber , F. Chisari , Immunity 1996, 4, 25. 8574849 10.1016/s1074-7613(00)80295-2

[54] L. Huang , H. Wu , P. Chen , D. Chen , Proc Natl Acad Sci U S A 2006, 103, 17862. 17095599 10.1073/pnas.0608578103 PMC1635544

[55] Y. Chen , X. Xiang , R. Qi , Y. Wang , Y. Huang , M. You , Y. Xian , Y. Wu , R. Fu , C. Kang , J. Tang , H. Yu , T. Zhang , Q. Yuan , W. Luo , N. Xia , Antib Ther 2021, 4, 197. 34646979 10.1093/abt/tbab020 PMC8499627

[56] B. Zhou , L. Xia , T. Zhang , M. You , Y. Huang , M. He , R. Su , J. Tang , J. Zhang , S. Li , Z. An , Q. Yuan , W. Luo , N. Xia , Antiviral Res. 2020, 180, 104757. 32171857 10.1016/j.antiviral.2020.104757

[57] A. Bradbury , J. Marks , J. Immunol. Methods 2004, 290, 29. 15261570 10.1016/j.jim.2004.04.007

[58] Y. Chen , W. Luo , W. Wu , Z. Fang , L. Xia , X. Gui , Y. Chen , H. Chen , J. Shih , N. Xia , Antiviral Res. 2010, 87, 81. 20450935 10.1016/j.antiviral.2010.04.012

