# Supplementary material for: A Dual‐domain Engineered Antibody for Efficient HBV Suppression and Immune Responses Restoration
Source: Adv Sci (Weinh). 2024 Feb 11;11(15):2305316. doi: 10.1002/advs.202305316 (PMC11022716; doi:10.1002/advs.202305316)
Supplement: Supplementary file 1 — Supporting Information [file ADVS-11-2305316-s001.pdf]

ADVANCED SCIENCE
Open Access

Supporting Information

for *Adv. Sci.*, DOI 10.1002/advs.202305316

A Dual-domain Engineered Antibody for Efficient HBV Suppression and Immune Responses Restoration

Yichao Jiang, Xiaoqing Chen, Xinya Ye, Can Wen, Tao Xu, Chao Yu, Wenjing Ning, Guosong Wang, Xinchu Xiang, Xiaomin Liu, Yalin Wang, Yuanzhi Chen, Xue Liu, Changrong Shi, Chao Liu, Quan Yuan, Yixin Chen, Tianying Zhang, Wenxin Luo* and Ningshao Xia**

Supplementary Information for

A Dual-domain Engineered Antibody for Efficient HBV Suppression and Immune Responses Restoration

Yichao Jiang[a, †], *Xiaoqing Chen*[a, †], *Xinya Ye*[a, †], *Can Wen*[a], *Tao Xu*[a], *Chao Yu*[a], *Wenjing Ning*[a], *Guosong Wang*[a], *Xinchu Xiang*[a], *Xiaomin Liu*[d], *Yalin Wang*[a], *Yuanzhi Chen*[a], *Xue Liu*[a], *Changrong Shi*[d], *Chao Liu*[d], *Quan Yuan*[a,b], *Yixin Chen*[a,b], *Tianying Zhang*[a,b,]*, *Wenxin Luo*[a,b,]*, *Ningshao Xia*[a,b,c,]*

Affiliations

[a]State Key Laboratory of Vaccines for Infectious Diseases, Xiang An Biomedicine Laboratory, School of Life Sciences, School of Public Health, Xiamen University, Xiamen 361102, PR China

[b]State Key Laboratory of Molecular Vaccinology and Molecular Diagnostics, National Institute of Diagnostics and Vaccine Development in Infectious Diseases, National Innovation Platform for Industry-Education Integration in Vaccine Research, School of Public Health, School of Life Sciences, Xiamen University, Xiamen 361102, PR China

[c]The Research Unit of Frontier Technology of Structural Vaccinology of Chinese Academy of Medical Sciences, Xiamen University, Xiamen 361102, PR China

[d]State Key Laboratory of Vaccines for Infectious Diseases, Center for Molecular Imaging and Translational Medicine, Xiang An Biomedicine Laboratory, School of Public Health, Xiamen University, Xiamen 361102, PR China

[†]These authors contributed equally to this work.

*Corresponding authors: Ningshao Xia (nsxia@xmu.edu.cn), Wenxin Luo (wxluo@xmu.edu.cn), Tianying Zhang (zhangtianying@xmu.edu.cn).

Keywords: Chronic hepatitis B; Antibody-based immunotherapy; Therapeutic efficacy; Immune restoration

Supplementary Figures

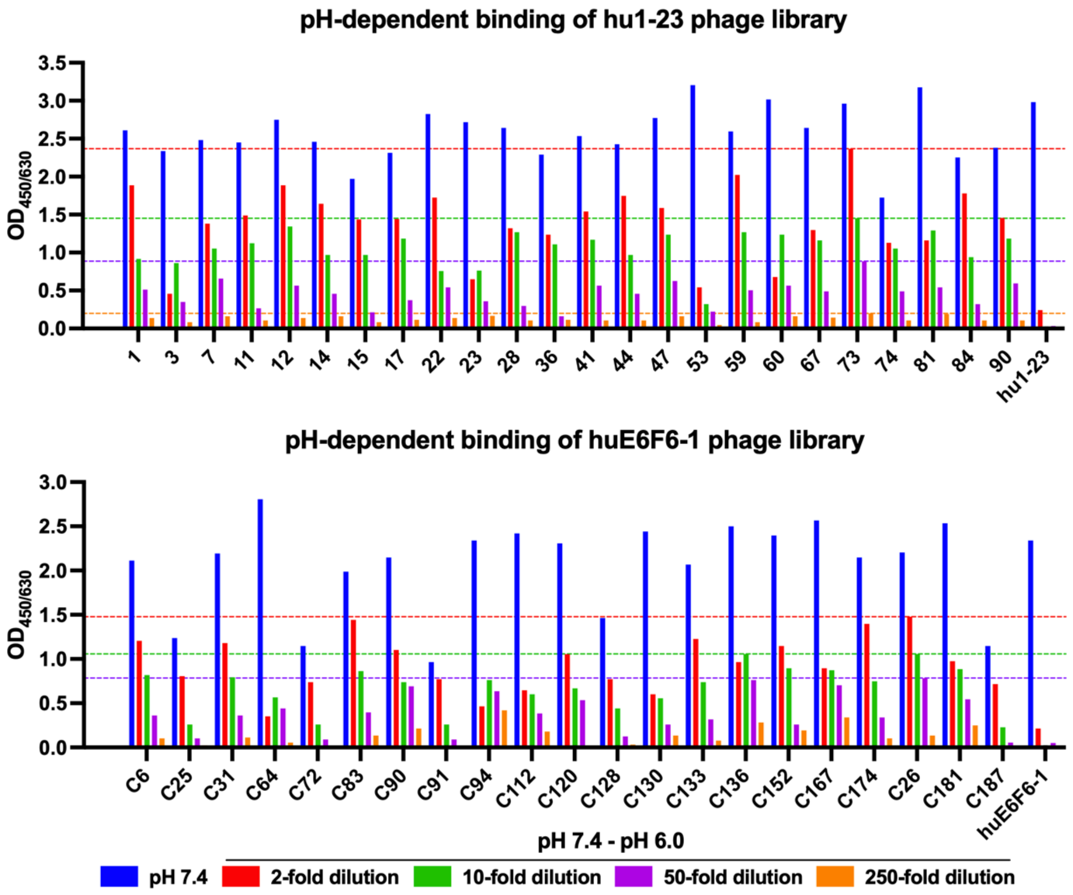

Figure S1. Screening of pH-dependent scFv-phage libraries and characterization of pH-dependent HBsAg-binding clones. (A) A schematic diagram indicating the procedure of construction and screening of pH-dependent scFv-phage libraries and expression of full-length antibodies. This figure was created with *BioRender.com*. (B) Determination of pH-dependent positive phage clones by ELISA analysis. Blue columns represent binding activity against HBsAg at pH 7.4. Red, green, violet and orange columns represent the differences between absorbance at pH 7.4 and pH 6.0, and phage supernatants were 5-fold serially diluted respectively. The dashed lines show the binding difference under indicated pH conditions and dilutions represented by 73 and C26.

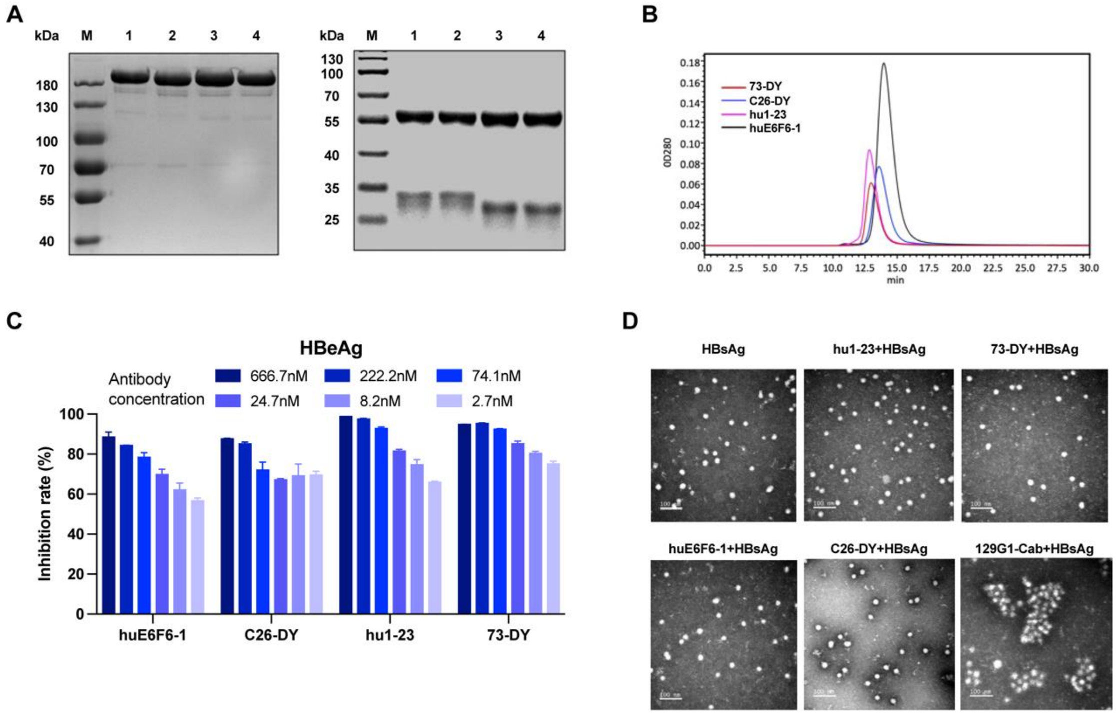

Figure S2. Characterization of 73-DY and C26-DY. (A) SDS-PAGE analysis of the Protein A purified antibodies under non-reducing (left) or reducing (right) conditions. Lane M, protein markers; lane 1, huE6F6-1; lane 2, C26-DY; lane 3, hu1-23; lane 4, 73-DY. (B) SEC-HPLC analysis of hu1-23, 73-DY, huE6F6-1 and C26-DY. (C) Neutralization of HBV infection in the hNTCP-expressed cell line by anti-HBV antibodies (n = 3). The levels of HBeAg were used to evaluate the HBV neutralization activity of the antibodies. The data were normalized to the virus infection control, and expressed as the mean ± SD. (D) TEM images of the antibody–HBsAg ICs formed by hu1-23, 73-DY, huE6F6-1, C26-DY and 129G1-Cab. 129G1-Cab is a chimeric antibody that recognizes the ‘second loop’ linear epitope (aa 137–151) of HBsAg and could induce viral particle aggregation.[1] Representative images from random fields of view in one of the three biologically independent samples. Scale bar: 100 nm.

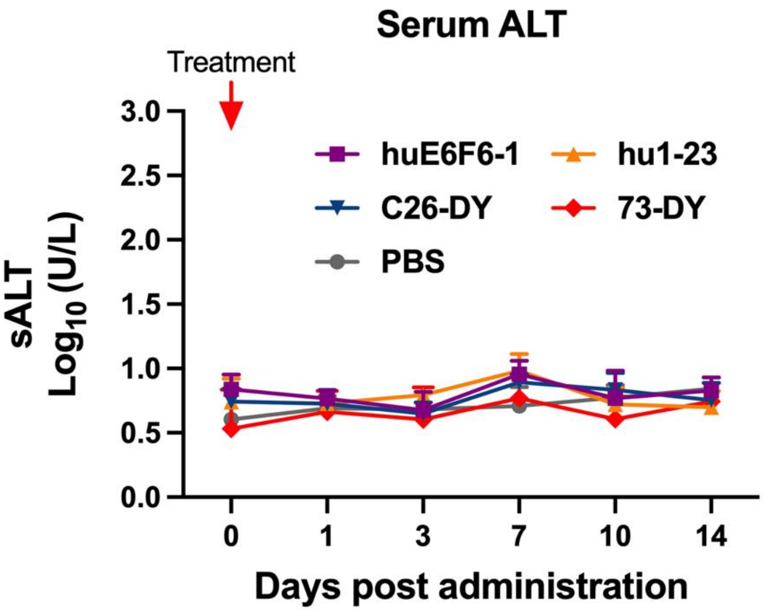

Figure S3. Serum ALT levels of AAV/HBV mice after antibody or PBS (Control) treatment (n = 4). The data are expressed as the mean ± SD.

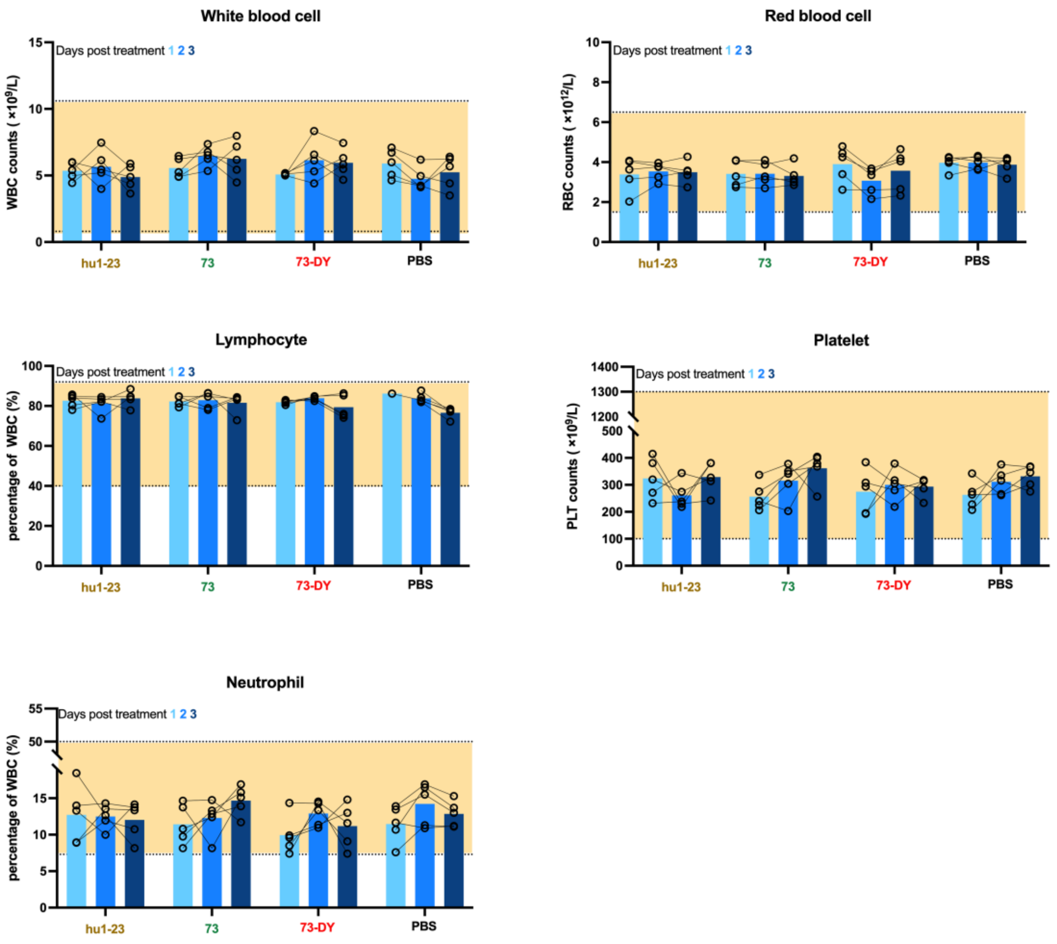

Figure S4. Blood routine examination of mice (*n* = 4) receiving antibody (hu1-23, 73, and 73-DY) or PBS (Control) on day 1-3 after treatment. Blood samples were analyzed by BC-5300VET Hematology Analyzer. The counts of white blood cell, red blood cell and platelet and the percentages of lymphocyte and neutrophil in white blood cell were calculated. The horizontal dotted lines and the yellow-shaded areas indicate the reference ranges of normal values provided by BC-5300VET Hematology Analyzer.

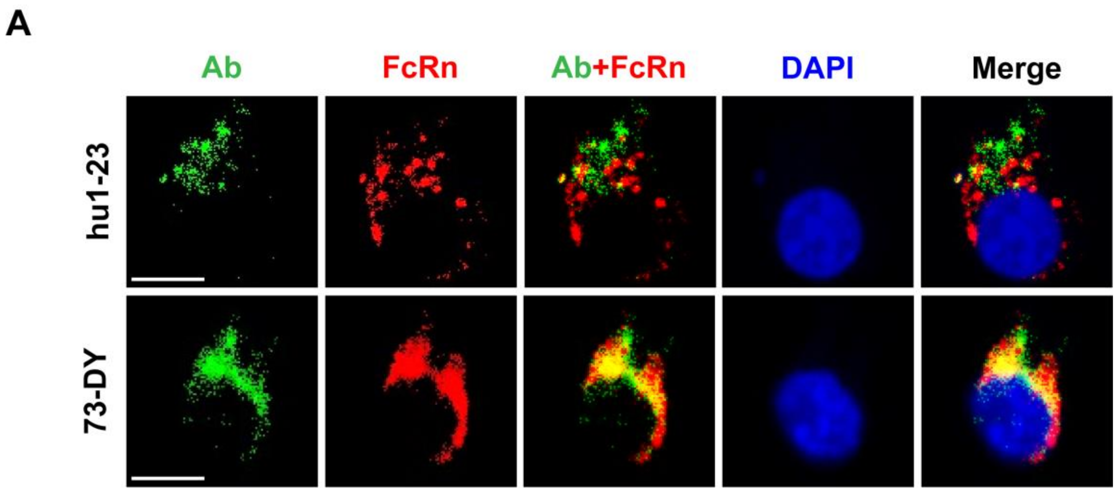

Figure S5. Confocal microscopy images of Raw264.7 cells incubated with hu1-23- or 73-DY-HBsAg immune complexes to show distribution of antibody and FcRn. hu1-23 or 73-DY was pre-incubated with recombinant HBsAg for 60 min, and then added to Raw264.7 cells for a further 60 min. Cells were washed, fixed and permeabilized. Antibodies (green) were labeled with DyLight 488-labeled mouse anti-human IgG (H+L) secondary antibody. FcRn (red) was labeled with goat anti-mouse FcRn followed by DyLight 568-labeled donkey anti-goat IgG. The co-localization between antibodies and FcRn is shown in yellow (overlap of green and red). Representative images from random fields of view in one of the three biologically independent samples. Scale bar: 10 μm.

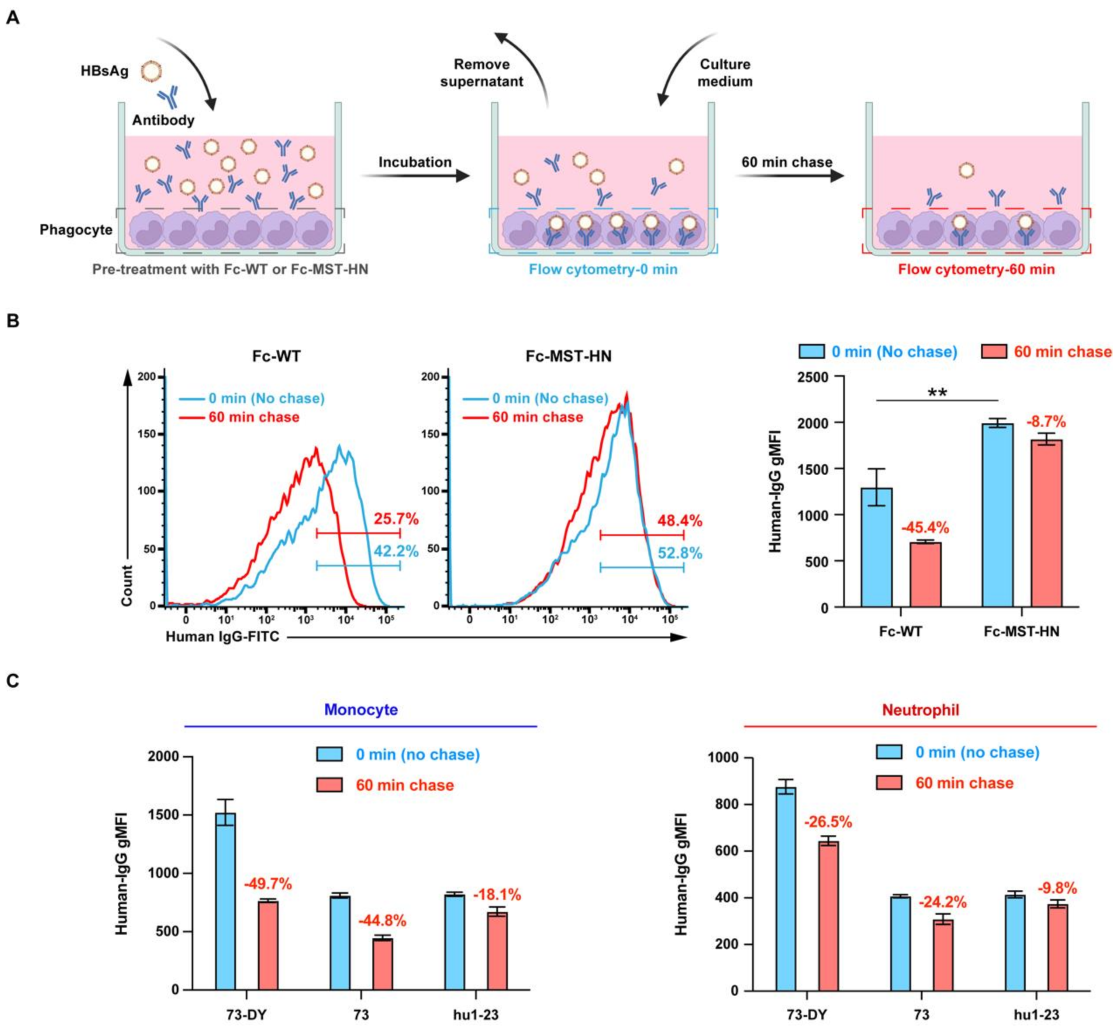

Figure S6. The antibody recycling study in phagocytes. (A) Schematic representation of the antibody recycling study. Phagocytes were pre-treated with wild-type Fc (Fc-WT) or MST-HN variant (M252Y/S254T/T256E/H433K/N434F, Fc-MST-HN; FcRn blocker) for 60 min. Subsequently, antibody-HBsAg immune complexes were added to cells for 60 min. Cells were then washed and processed immediately (no chase) or incubated in fresh RPMI culture medium for a further 60 min. After each treatment, cells were washed, fixed and permeabilized, and subsequently stained with DyLight 488-labeled anti-human IgG (H+L) secondary antibody. Percentages of human-IgG-FITC positive cells and geometric mean fluorescence intensities (gMFI) of FITC fluorescence at each time point were quantified by flow cytometry. (B) Flow cytometric analysis of Raw264.7 cells at different chase times after pretreatment with either Fc-WT of Fc-MST-HN and subsequent incubation with 73-DY-HBsAg immune complexes (n = 3). Histogram plots (left) are representative from three biologically

independent samples, and the gMFI data (right) are shown as the mean ± SD. *P* values were calculated using a two-sided Student's *t*-test (**$P < 0.01$). (C) Antibody recycling study in monocytes and neutrophils. In this assay, cells were not pre-treated with either Fc-WT or Fc-MST-HN. Different ICs were added to monocytes and neutrophils, respectively ($n = 3$). gMFI of FITC fluorescence at each time point was quantified by flow cytometry, and the data are expressed as the mean ± SD. For (B) and (C), the reductions in the human-IgG gMFI after 60-min chase were calculated and marked in red font.

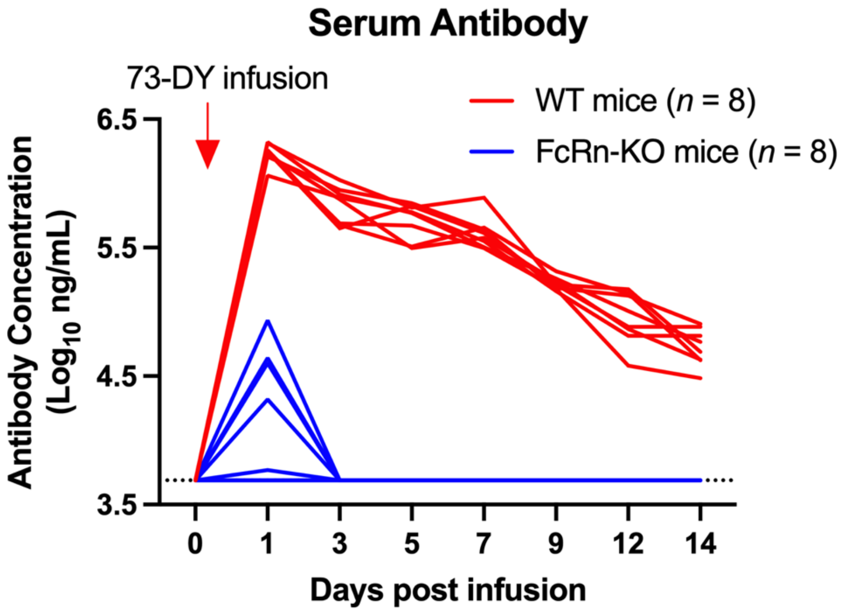

Figure S7. Pharmacokinetics study of 73-DY in AAV/HBV infected wild-type (WT) mice or FcRn knockout (FcRn-KO) mice. Each group of mice (n = 8) received 73-DY infusion at a dose of 10 mg/kg. Individual antibody concentration curves are shown. The horizontal dotted line indicates the lowest detection limit.

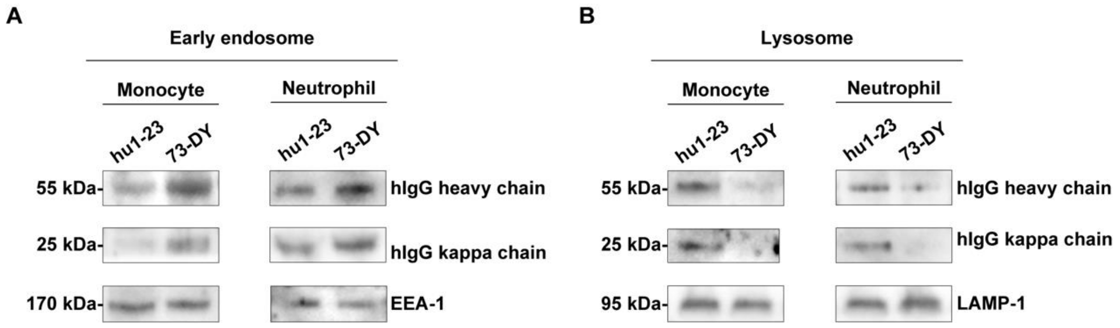

Figure S8. Detection of antibody components in the early endosomes or lysosomes of phagocytes from antibody-treated AAV/HBV mice by western blotting analysis. The monocytes and neutrophils were obtained from the peripheral blood of AAV/HBV mice one day after administration of hu1-23 or 73-DY at a dose of 10 mg/kg. Early endosomes (A) or lysosomes (B) were extracted from monocytes and neutrophils using specific isolation kits and subsequently lysed. The presence of antibody components, including hIgG heavy chains and kappa chains, in the lysates were assessed using HRP-conjugated anti-human IgG (H+L) secondary antibody. Due to the absence of GAPDH or β-actin in early endosomes or lysosomes, early endosome antigen-1 (EEA-1) or lysosome associated membrane protein-1 (LAMP-1) was used as a loading control for early endosome or lysosome samples, respectively. Representative results of three biologically independent samples with similar results are shown. The samples derive from the same experiment and that gels/blots were processed in parallel.

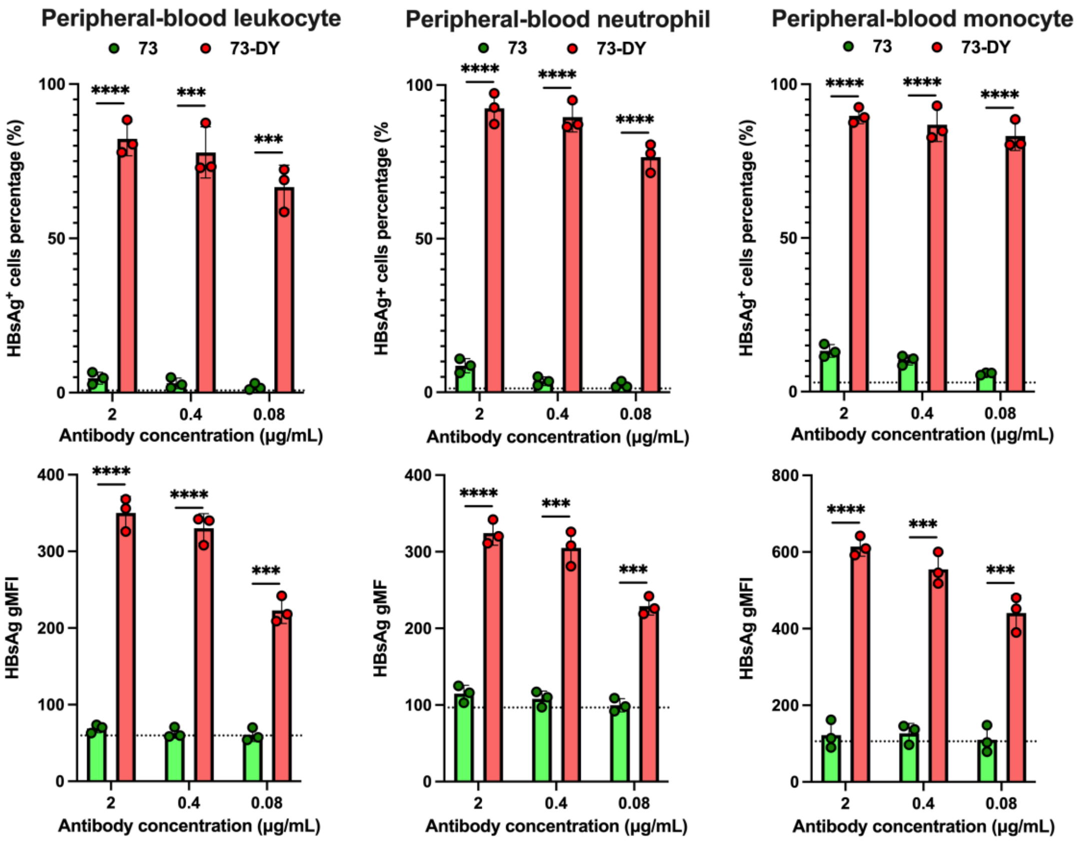

Figure S9. Flow cytometric analyses of 73- and 73-DY-mediated *ex vivo* phagocytosis by phagocytes from peripheral blood. Neutrophils and monocytes from peripheral blood were identified by specific antibodies. The percentage of $HBsAg^{+}$ cells and the HBsAg gMFI were calculated, and the horizontal dotted lines indicate the percentage of $HBsAg^{+}$ cells and the HBsAg gMFI for spontaneous HBsAg phagocytosis without antibody treatments ($n = 3$). The P values were calculated using a two-sided Student's t-test (***$P < 0.001$; ****$P < 0.0001$).

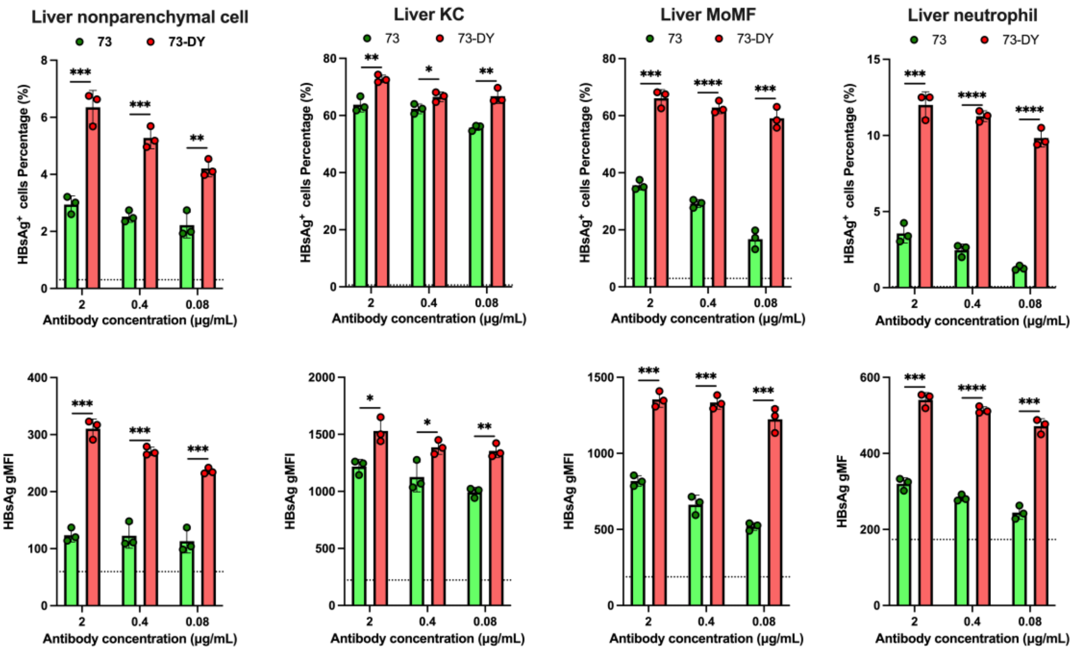

Figure S10. Flow cytometric analyses of 73- and 73-DY-mediated *ex vivo* phagocytosis by phagocytes from liver nonparenchymal cells. KCs, monocyte-derived macrophages (MoMFs) and neutrophils from liver nonparenchymal cells were identified by specific antibodies. The percentage of $HBsAg^+$ cells and the HBsAg gMFI were calculated, and the horizontal dotted lines indicate the percentage of $HBsAg^+$ cells and the HBsAg gMFI for spontaneous HBsAg phagocytosis without antibody treatments (n = 3). The P values were calculated using a two-sided Student's t-test (*P < 0.05; **P < 0.01; ***P < 0.001; ****P < 0.0001).

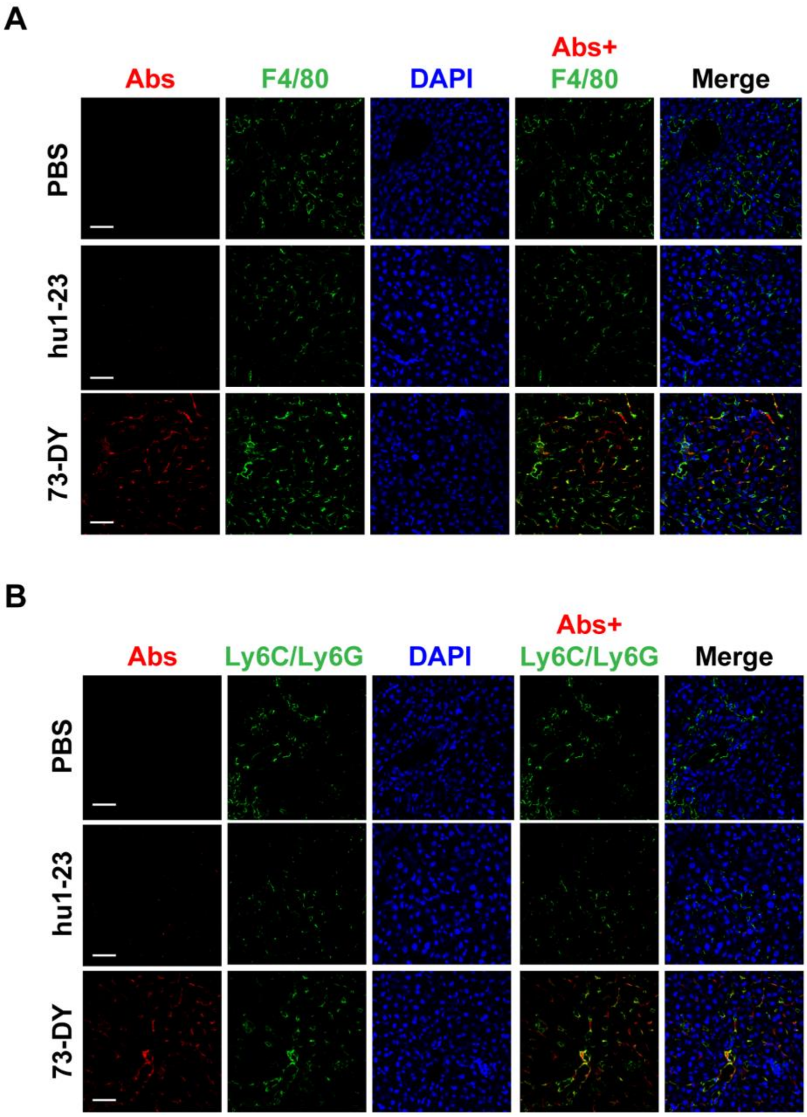

Figure S11. Immunofluorescence staining of liver sections of AAV/HBV mice after treatment with hu1-23, 73-DY or PBS. Assays were performed 6 days after antibody or PBS infusion. Antibodies (red) were labeled with DyLight 650-labeled anti-human IgG, and liver phagocytes (green) were labeled with rat anti-mouse F4/80 (A) or rat anti-mouse Ly6C/Ly6G (B) followed by DyLight 568-labeled anti-rat IgG. The co-localization between antibodies and F4/80 or Ly6C/Ly6G is shown in yellow (overlap of green and red). Representative images from random fields of view in one of the three biologically independent samples. Scale bar: 40 μm.

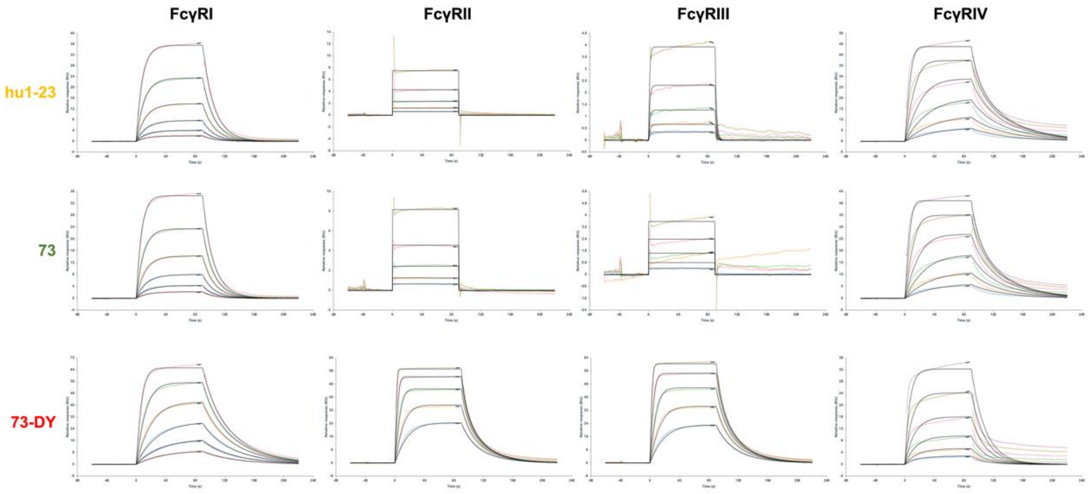

Figure S12. The affinity of hu1-23, 73 and 73-DY for various classes of murine FcγRs was determined by surface plasmon resonance (SPR). The SPR sensorgrams are representative of three biologically independent experiments.

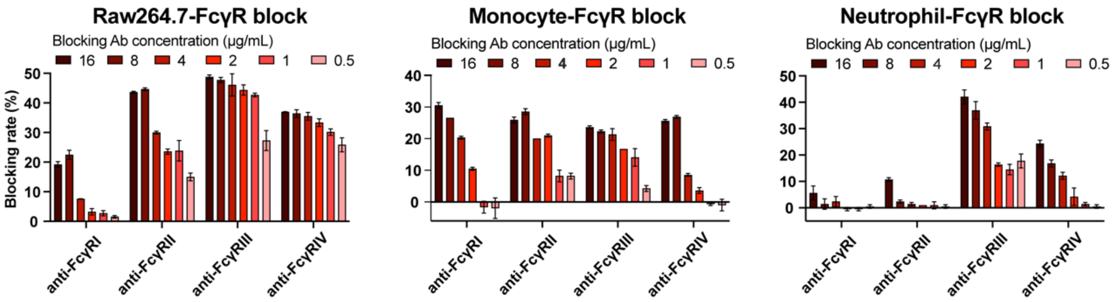

Figure S13. Specific FcγR blocking of the 73-DY-mediated phagocytosis of HBsAg in Raw264.7 cells, murine monocytes and neutrophils. Cells were evaluated by flow cytometric analysis, and the HBsAg gMFI were calculated (n = 3). The blocking rate was calculated with the following formula: [(gMFI of 73-DY-mediated phagocytosis without FcγR blocking – gMFI of 73-DY-mediated phagocytosis with indicated FcγR blocking) / (gMFI of 73-DY-mediated phagocytosis without FcγR blocking – gMFI of spontaneous phagocytosis by indicated phagocytes)] × 100%. The data are expressed as the mean ± SD.

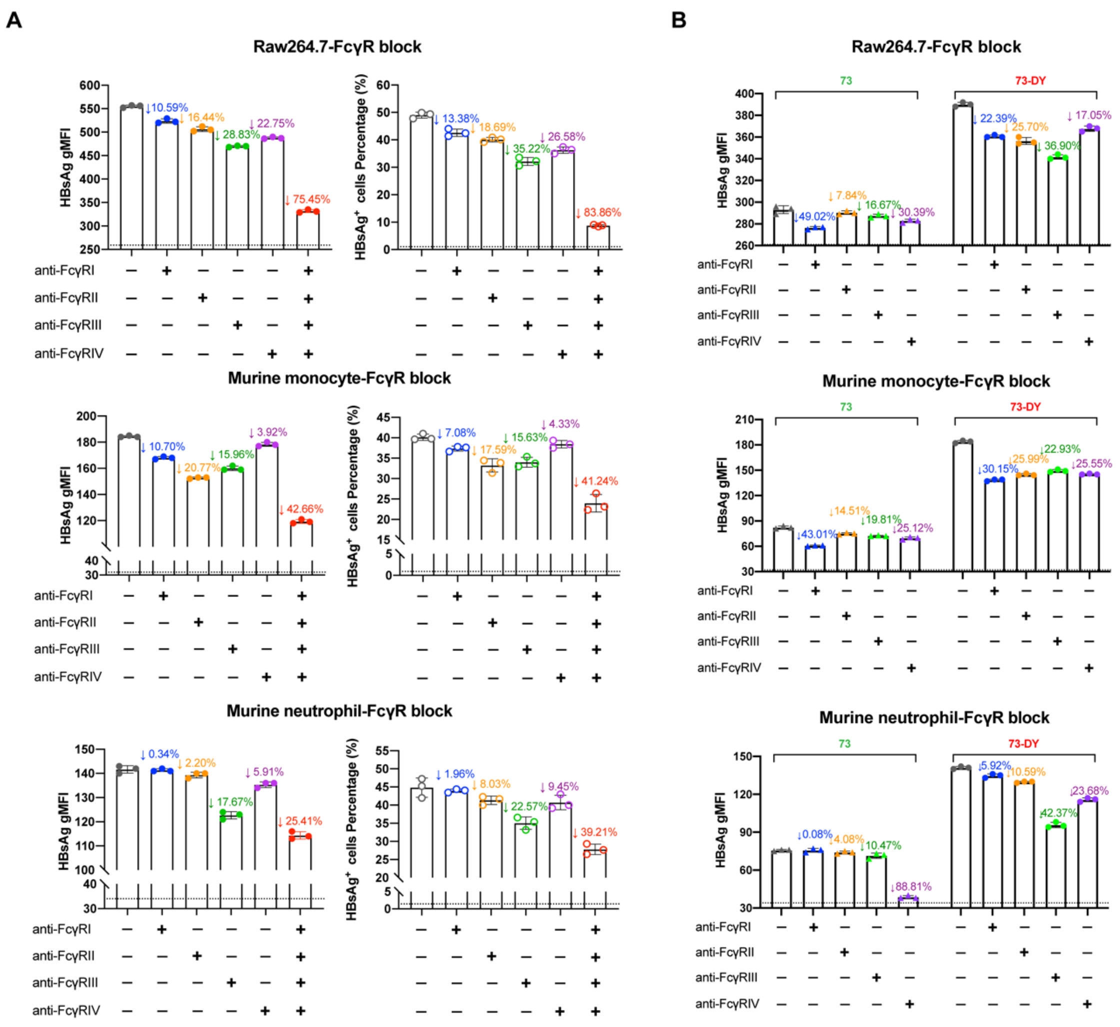

Figure S14. The reduction of antibody-dependent cellular phagocytosis of HBsAg by FcγR-blocking. (A) The reduction of 73-DY-mediated ADCP by 2 μg/mL of anti-FcγR blocking antibody alone or combination (n = 3). The percentage of HBsAg$^+$ cells and HBsAg gMFI were calculated and the horizontal dotted lines indicate the data provided by spontaneous HBsAg phagocytosis without 73-DY treatment. The data are expressed as the mean ± SD. The blocking rate was calculated with the following formula: [(gMFI of 73-DY-mediated phagocytosis without FcγR blocking – gMFI of 73-DY-mediated phagocytosis with indicated FcγR blocking) / (gMFI of 73-DY-mediated phagocytosis without FcγR blocking – gMFI of spontaneous phagocytosis by indicated phagocytes)] × 100%. (B) The reduction of 73- or 73-DY-mediated ADCP by anti-FcγR blocking antibody alone at the concentration of 16 μg/mL, which could achieve complete blocking (n = 3). The HBsAg gMFI was calculated and the horizontal dotted lines indicate the data provided by spontaneous HBsAg phagocytosis without 73

or 73-DY treatment. The data are expressed as the mean ± SD. The blocking rates in the HBsAg gMFI after specific FcγR blocking were calculated and presented.

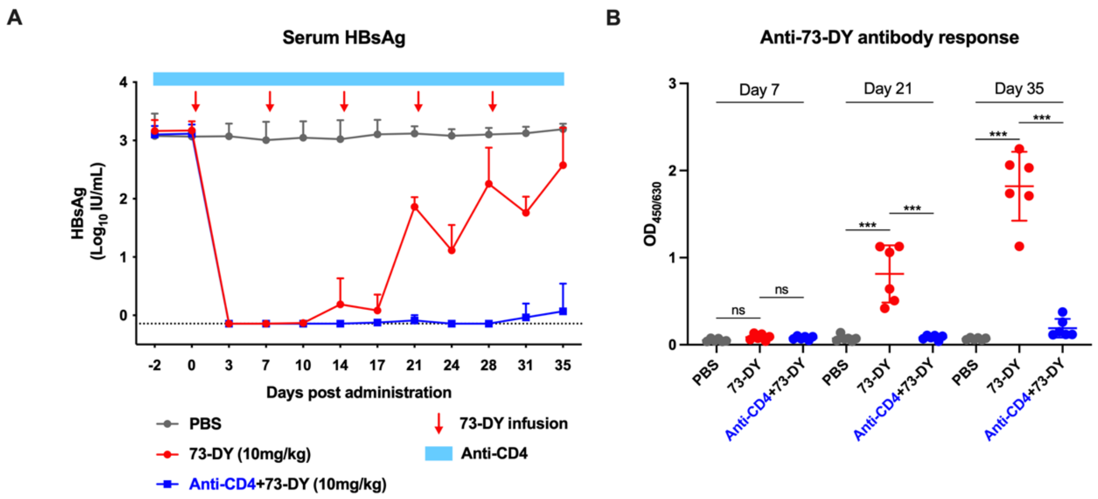

Figure S15. The efficacy of repeated injection of 73-DY and associated anti-drug antibody responses *in vivo*. (A) Serum HBsAg levels of AAV/HBV mice treated with 73-DY or PBS (Control) infusion at a dose of 10 mg/kg once weekly (n = 6 mice per group). By the injection of 150 μg of anti-mouse CD4 (clone GK1.5) two days before treatment initiation and then 100 μg every other day, the impaired 73-DY efficacy was rescued. The data are presented as the mean ± SD. The horizontal dotted line indicates the lowest detection limit. (B) Anti-73-DY antibody responses evaluated by ELISA analysis. Serum samples collected from the mice mentioned in (A) at indicated time points were diluted by 100-fold and added to pre-coated 73-DY plates (n = 6). The presence of mouse anti-73-DY antibodies was detected using HRP-conjugated anti-mouse IgG (H+L) secondary antibody. The anti-CD4 administration greatly eliminated the production of anti-HBs. The data are expressed as the mean ± SD. The statistical significance was determined using a two-sided Student's t-test (***P < 0.001; "ns" represents not significant).

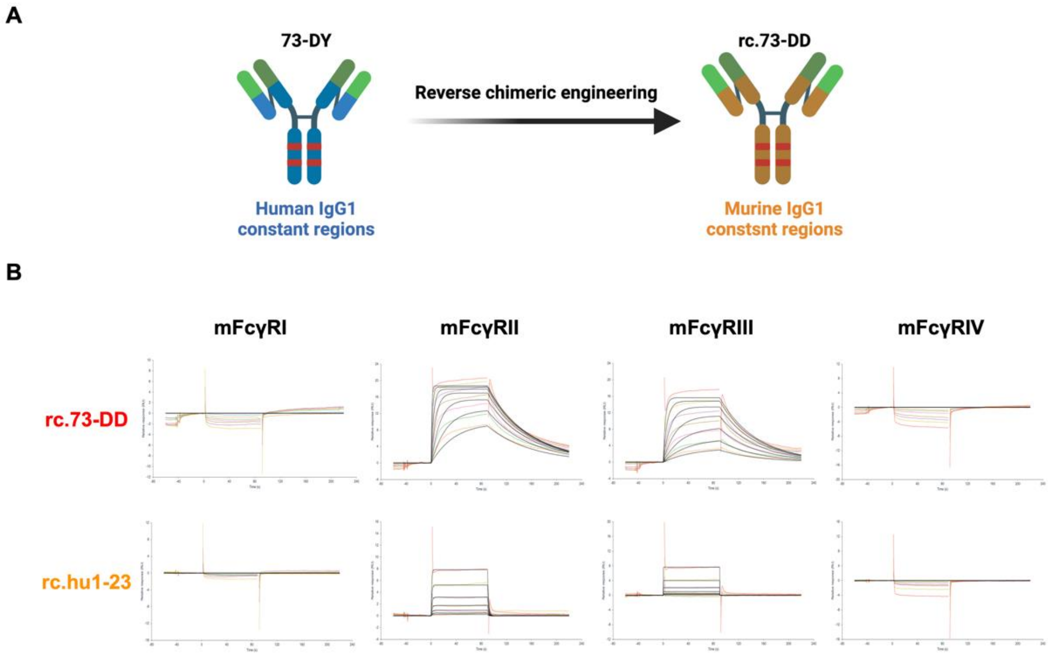

Figure S16. The generation and characterization of reverse chimeric antibody rc.73-DD. (A) Schematic representation of the generation of rc.73-DD. The variable regions of 73-DY were introduced to the N-terminals of the murine IgG1 constant regions with Fc mutations of S239D/A327D. This figure was created with *BioRender.com*. (B) The affinity of rc.hu1-23 and rc.73-DD for various classes of murine FcγRs was determined by SPR. The SPR sensorgrams are representative of three biologically independent experiments.

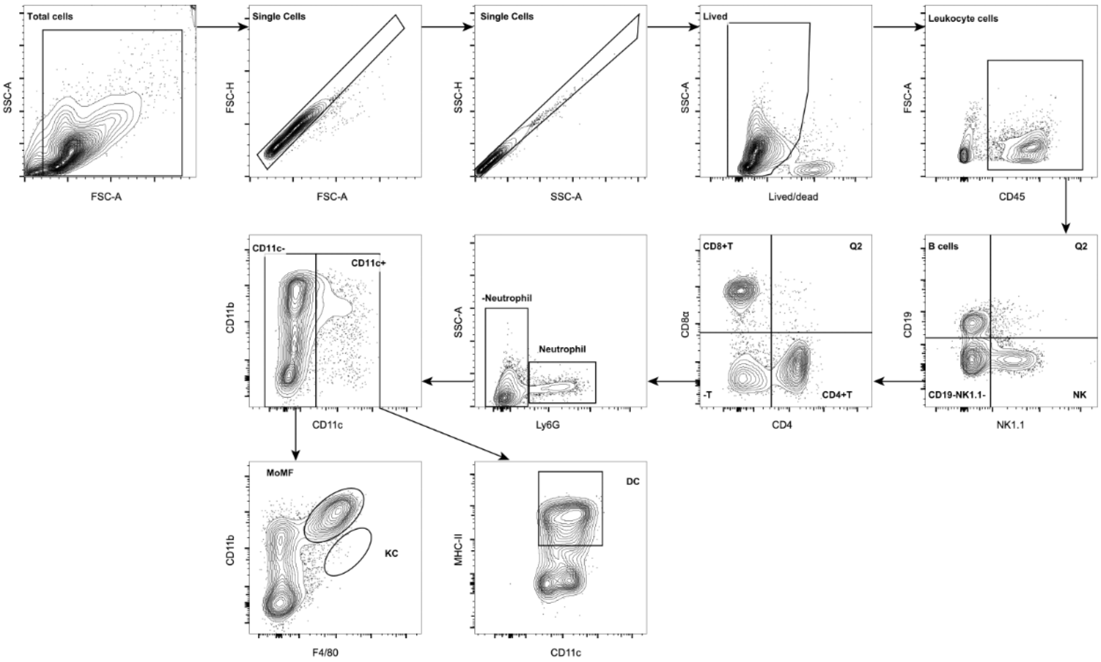

Figure S17. Gating strategy of flow cytometric analysis. Identification of various leukocyte populations. Representative images from one of six biologically independent samples are shown.

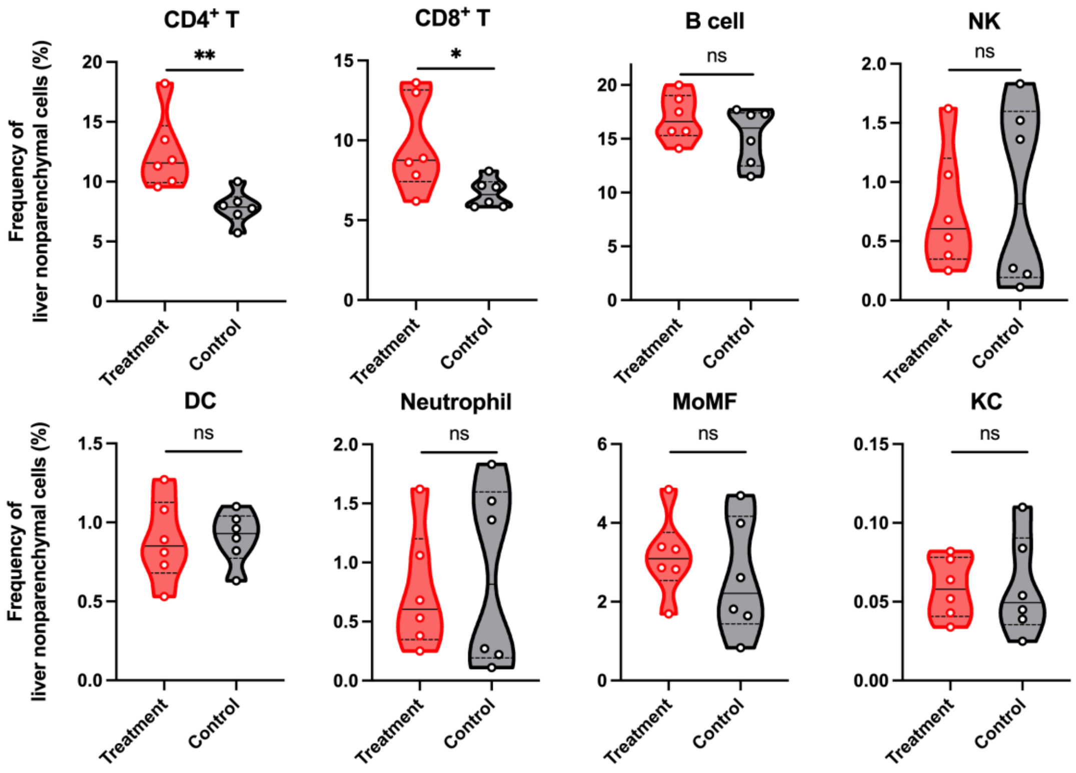

Figure S18. Flow cytometric analysis of the immune responses of rc.73-DD-treated group and PBS-treated (Control) group. Frequencies of typical immune cells in the liver nonparenchymal cells (n = 6). Data were statistically analyzed by a two-sided Student's t-test (*$P < 0.05$; **$P < 0.01$; "ns" represents not significant).

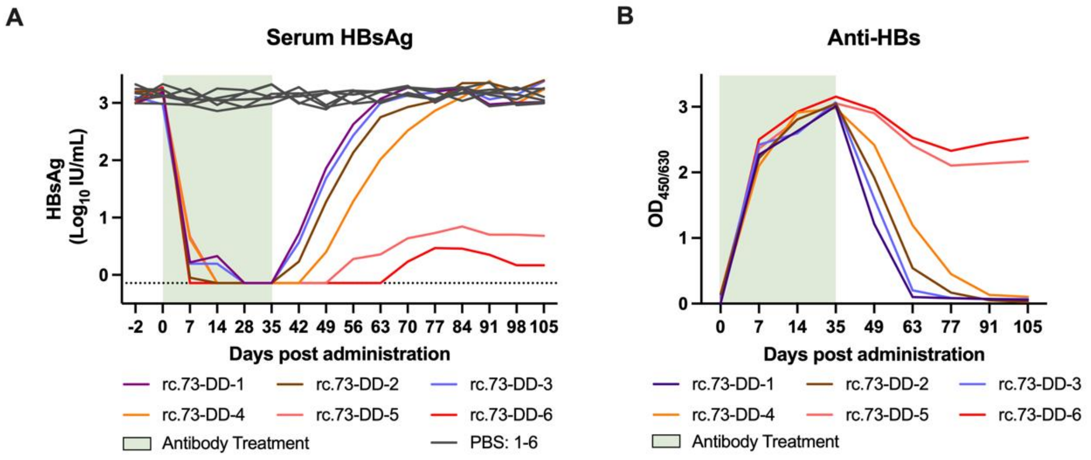

Figure S19. The efficacy of repeated injection of reverse chimeric 73-DY *in vivo*. (A) Serum HBsAg levels of AAV/HBV mice treated with rc.73-DD or PBS (Control) infusion at a dose of 20 mg/kg twice weekly (*n* = 6 mice per group). Individual HBsAg titer curves are shown. The horizontal dotted line indicates the lowest detection limit. (B) Anti-HBsAg antibody responses assessed by ELISA analysis. Serum samples collected from the rc.73-DD-treated mice mentioned in (A) at indicated time points were diluted by 50-fold and added to pre-coated HBsAg plates (*n* = 6). The presence of mouse anti-HBsAg antibodies was detected using HRP-conjugated anti-mouse IgG (H+L) secondary antibody. Individual anti-HBs curves are shown.

Supplementary Tables

Table S1. The strategies and results of four rounds of scFv-phage library screening for pH-dependently HBsAg binding.

**Panning rounds**	**1st round**		**2nd round**		**3rd round**				**4th round (Harsh)**			
**Batch NO.**	**M1-A1**	**M1-B1**	**M1-A2**	**M1-B2**	**M1-A31**	**M1-A32**	**M1-B31**	**M1-B32**	**M1-A41**	**M1-A42**	**M1-A43**	**M1-A44**
**Coated antigen HBsAg concentration (ng/well)**	**200**		**200**		**200**				**20**	**20**	**100**	**100**
**Incubation time**	**30 min**		**30 min**		**10 min**				**5 min**			
**Washing reagent (washing times)**	**PBS (4)**		**PBS (8), PBST (3)**		**PBS (8), PBST (3)**	**PBS (12), PBST (8) 4M MgCl2 30 min**	**PBS(8), PBST (3)**	**PBS (12), PBST (8) 4M MgCl2 30 min**	**PBS (12), PBST (8)**			

						50 mM NaOH		50 mM NaOH				
**Eluent pH**	**4.0**		**5.0**		**6.0**				**6.0**	**6.5**	**6.0**	**6.5**
**Soaking time**	**0**	**30 min**	**0**	**30 min**	**0**	**0**	**30 min**	**30 min**	**0**			
**Input (cfu)**	**5×10^{12}**	**5×10^{12}**	**5×10^{12}**	**5×10^{12}**	**1×10^{11}**	**1×10^{11}**	**1×10^{11}**	**1×10^{11}**	**1×10^{11}**			
**Output (cfu)**	**7×10^{7}**	**5×10^{6}**	**7×10^{6}**	**1×10^{7}**	**1×10^{6}**	**1×10^{5}**	**1×10^{6}**	**2×10^{5}**	**370**	**480**	**1004**	**277**
**HBsAg-specific binding clone positive rate**					**96%**	**80%**	**77%**	**98%**	**71%**	**48%**	**85%**	**76%**
**pH-dependently HBsAg binding clone positive rate**					**9%**	**0**	**11%**	**0**	**72%**	**72%**	**50%**	**48%**

Table S2. pH-dependent HBsAg-binding properties of antibodies.

Antibodies	Maximal-OD450/630		Maximal-OD450/630 ratio pH 6.0 / pH 7.4	EC50 (ng/mL)		EC50 ratio pH 6.0 / pH 7.4	EC50 (pH 7.4) ratio vs. WT
	pH 6.0	pH 7.4		pH 6.0	pH 7.4		
**hu1-23**	3.982	4.022	0.99	35.43	38.78	0.91	-
**73**	1.139	3.759	0.30	2885	128.7	22.42	3.74
**huE6F6-1**	4.050	3.914	1.03	25.65	21.98	1.17	-
**C26**	2.014	3.897	0.52	153.8	85.16	1.81	5.96

EC50 values were calculated from the nonlinear regression best-fit curves in Figure 1B by GraphPad Prism (v.9.0).

Table S3. CDRs and mutation summary of Fab-engineered antibodies 73 and C26.

Antibodies	HCDR1	HCDR2	HCDR3	LCDR3
hu1-23	GGSITSNFW	SGSGTYT	ARSHDYGSNDYAFDF	QQYHSLPLT
73	* * * *H* * * *	* * P*HH*	* * * * * * *HH* * * * * *	* * * *Y* * * *
huE6F6-1	YGYHWN	YISYDGSVLYNPSLEN	GFDH	SQNTHVPYT
C26	H * * * * *	*LH* * * * * * * * * * * * *	* * * *	* * * * *H* * *

Table S4. Characterization of the affinities of rc.hu1-23 and rc.73-DD for murine FcγRs via BIAcore analysis.

Antibody	FcγRI		FcγRII		FcγRIII		FcγRIV	
	K_D (M)	Fold	K_D (M)	Fold	K_D (M)	Fold	K_D (M)	Fold
**rc.hu1-23**	N.D.	-	1.96E-07	1.00	2.68E-06	1.00	N.D.	-
**rc.73-DD**	N.D.	-	2.97E-09	65.99	1.18E-08	227.12	N.D.	-

The fold change was calculated in affinity compared with that of rc.hu1-23. N.D., not detectable.

References

1. Zhang, T.Y., Q. Yuan, J.H. Zhao, Y.L. Zhang, L.Z. Yuan, Y. Lan, Y.C. Lo, C.P. Sun, C.R. Wu, J.F. Zhang, Y. Zhang, J.L. Cao, X.R. Guo, X. Liu, X.B. Mo, W.X. Luo, T. Cheng, Y.X. Chen, M.H. Tao, J.W.K. Shih, Q.J. Zhao, J. Zhang, P.J. Chen, Y.A. Yuan, N.S. Xia, Prolonged suppression of HBV in mice by a novel antibody that targets a unique epitope on hepatitis B surface antigen. *Gut*, 2016. **65**(4): p. 658-671.
